# Sequence determinants of protein phase behavior from a coarse-grained model

**DOI:** 10.1371/journal.pcbi.1005941

**Published:** 2018-01-24

**Authors:** Gregory L. Dignon, Wenwei Zheng, Young C. Kim, Robert B. Best, Jeetain Mittal

**Affiliations:** 1 Department of Chemical and Biomolecular Engineering, Lehigh University, Bethlehem, Pennsylvania, United States of America; 2 Laboratory of Chemical Physics, National Institute of Diabetes and Digestive and Kidney Diseases, National Institutes of Health, Bethesda, Maryland, United States of America; 3 Center for Materials Physics and Technology, Naval Research Laboratory, Washington, DC, United States of America; Bar Ilan University, ISRAEL

## Abstract

Membraneless organelles important to intracellular compartmentalization have recently been shown to comprise assemblies of proteins which undergo liquid-liquid phase separation (LLPS). However, many proteins involved in this phase separation are at least partially disordered. The molecular mechanism and the sequence determinants of this process are challenging to determine experimentally owing to the disordered nature of the assemblies, motivating the use of theoretical and simulation methods. This work advances a computational framework for conducting simulations of LLPS with residue-level detail, and allows for the determination of phase diagrams and coexistence densities of proteins in the two phases. The model includes a short-range contact potential as well as a simplified treatment of electrostatic energy. Interaction parameters are optimized against experimentally determined radius of gyration data for multiple unfolded or intrinsically disordered proteins (IDPs). These models are applied to two systems which undergo LLPS: the low complexity domain of the RNA-binding protein FUS and the DEAD-box helicase protein LAF-1. We develop a novel simulation method to determine thermodynamic phase diagrams as a function of the total protein concentration and temperature. We show that the model is capable of capturing qualitative changes in the phase diagram due to phosphomimetic mutations of FUS and to the presence or absence of the large folded domain in LAF-1. We also explore the effects of chain-length, or multivalency, on the phase diagram, and obtain results consistent with Flory-Huggins theory for polymers. Most importantly, the methodology presented here is flexible so that it can be easily extended to other pair potentials, be used with other enhanced sampling methods, and may incorporate additional features for biological systems of interest.

## Author summary

Liquid liquid phase separation (LLPS) of low-complexity protein sequences has emerged as an important research topic due to its relevance to membraneless organelles and intracellular compartmentalization. However a molecular level understanding of LLPS cannot be easily obtained by experimental methods due to difficulty of determining structural properties of phase separated protein assemblies, and of choosing appropriate mutations. Here we advance a coarse-grained computational framework for accessing the long time scale phase separation process and for obtaining molecular details of LLPS, in conjunction with state of the art enhanced sampling methods. We are able to qualitatively capture the changes of the phase diagram due to specific mutations, inclusion of a folded domain, and variation of chain length. The model is flexible and can be used with different knowledge-based potential energy functions, as we demonstrate. We expect a wide application of the presented framework for advancing our understanding of the formation of liquid-like protein assemblies.

## Introduction

Intracellular compartmentalization is essential for normal physiological activity. This is commonly accomplished through isolation by lipid membranes or vesicles, but can also be achieved without the use of a membrane via membraneless organelles [1–3]. These organelles include processing bodies [4], stress granules [3, 5–7] and germ granules [8, 9] in the cytoplasm, and nucleoli [10] and nuclear speckles [11] in the nucleus. It has recently been established that many of these membraneless organelles can be described as phase separated liquid-like droplets [8, 12]. The process of liquid-liquid phase separation (LLPS) allows these organelles to spontaneously coalesce and disperse, and is important for many biological functions, such as response to heat shock and other forms of stress [6, 13, 14], DNA repair [15, 16], regulation of gene expression [17, 18], cellular signaling [3, 19], and many other functions requiring spatial organization and biochemical regulation [10, 20–22]. LLPS has also been implicated as a precursor to the formation of hydrogels [23] and fibrillar aggregates [7, 15], suggesting possible relevance to the pathogenesis of many diseases including Amyotrophic Lateral Sclerosis (ALS) and Frontotemporal Dementia (FTD) [15, 24].

Experimental studies have characterized different properties of biological LLPS, and have shown that many systems share several common characteristics. First, the formation and dissolution processes can be tuned by the cellular environment such as changes in temperature, pH and salt concentration [25], by post-translational modification such as phosphorylation [19, 26], and by mixing with other biomolecules such as proteins [27], RNA [28–30], and ATP [2, 30]. Second, the concentrated phase has liquid-like properties, including fusion, dripping, wetting [25] and Ostwald ripening [28], and its viscosity is typically several orders of magnitude higher than that of water [2, 8, 25]. Third, LLPS is commonly driven or modulated by low complexity (LC) intrinsically disordered regions (IDRs) of the protein sequence [6, 25, 31], suggesting similarities to the well-characterized LLPS of polymer mixtures [32]. It should be noted that a disordered domain is not necessary for LLPS to occur [14], and indeed LLPS is known to occur for folded proteins during crystallization or purification [33]. Folded domains along with IDRs have also been shown to modulate LLPS properties [34]. Lastly, some proteins involved in LLPS process are also able to form fibril structures [7, 15], suggesting a possible connection between the liquid-like droplet and solid fibril states. However, the molecular level understanding of LLPS cannot be easily obtained by experimental methods due to the difficulty of obtaining structural properties even in the concentrated phase [6], and the cumbersome process of screening mutations [35].

A number of recent theoretical and simulation studies have addressed protein phase separation. Jacobs and Frenkel used Monte Carlo simulations to study multiple-component phase separation and found that the phase boundary is very sensitive to intermolecular interactions, but less dependent on the number of components in the system [36]. Lin and Chan applied the random phase approximation to treat electrostatic interactions [37] and Flory-Huggins theory for mixing entropy and other interactions. They were able to capture the sequence specificity of charged amino acids and found that the dependence of the phase boundary of the IDP Ddx4 on salt concentration can be explained by considering only electrostatic screening in their model [38]. It was also found that the monomer radius of gyration (*R*_*g*_) is correlated with the corresponding critical temperature in both theoretical work [39] and experiment [14]. This supports the hypothesis that fundamental polymer physics principles can be used to understand LLPS [40]. However, a computational framework capable of capturing the general sequence specificity including both hydrophobic and electrostatic interactions and molecular details on both intra- and inter-molecular interactions is still missing. All-atom simulation has the potential of fulfilling both tasks [41, 42] with the use of force fields suitable for intrinsically disordered proteins (IDPs) [43, 44]. Such a force field has been recently applied to study the monomer properties of TDP-43 which is known to undergo LLPS [45]. However, computational efficiency imposes limits on the use of all-atom representation for simulating LLPS directly. Even the use of coarse-grained simulations requires well-designed sampling methods to overcome the enthalpy gap between the two phases [46, 47].

In this work, we introduce a general computational framework for studying LLPS, combining a residue based potential capable of capturing the sequence specific interactions and the slab simulation method capable of achieving convergence for phase transition properties including critical temperature, and protein concentration in dilute and concentrated phases. To demonstrate the capabilities of the model, we have selected two model proteins: the LC domain of RNA-binding protein, Fused in Sarcoma (FUS), and the DEAD-box helicase protein, LAF-1, both of which are able to phase separate in vitro and in vivo [6, 15, 25]. Mutations of FUS have been shown to be highly relevant to the pathogenesis of ALS [48, 49] and display the ability to alter the kinetics of both droplet formation and aggregation into fibrils [15]. In addition, both the full length and disordered domain of LAF-1 phase separate in vitro [25], allowing us to explore the impact of a large, rigid domain on the LLPS behavior.

The manuscript is organized as follows. First, we introduce our computational framework including the coarse-grained potential, the sampling method and the treatment of folded proteins in the simulations. We then present the application of the method using two model systems. For the first system, we show the comparison of phase diagrams for wild-type (WT) FUS and a set of mutants, and that they are qualitatively consistent with recent experimental measurements. For the second, we demonstrate how inclusion of the folded domain alters the LAF-1 phase diagram. In both FUS and LAF-1, we show the flexibility of the framework by providing the results for two different coarse-grained potentials. Lastly, we investigate the phase diagram dependence on chain length, closely related to the “multivalency” effect often discussed in the context of LLPS.

## Methods

### Coarse-grained model development

**Fig 1:**
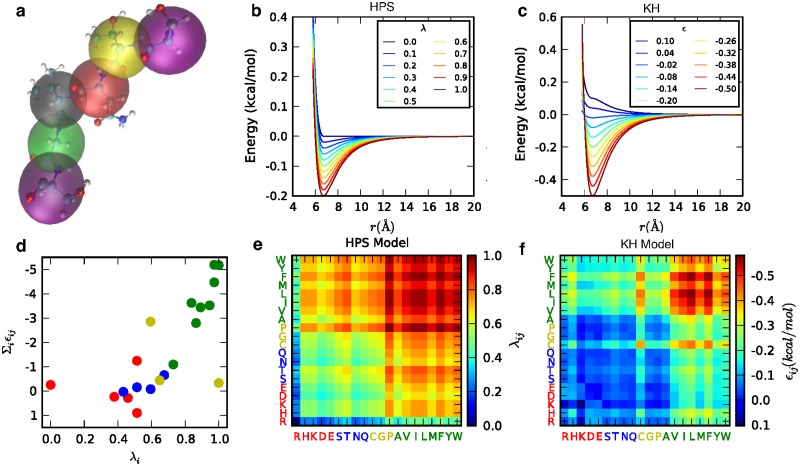
Schematic of the two knowledge-based potentials used for short-range pairwise interactions. a) Each amino acid is treated as a single particle. b, c) Potential energy functional form for HPS and KH models at different interaction strengths, plotted with a constant *σ* value of 6 Å. d) Correlation between the amino acid interaction strength (Σ_*i*_ *ϵ*_*ij*_) in KH model and hydrophobicity (*λ*_*i*_) in HPS model, colored by the side-chain properties of amino acids (i.e., red for charged, blue for polar, green for hydrophobic and yellow for other amino acids). e, f) The pairwise interaction parameters used in HPS and KH models shown in color maps with blue being most repulsive interactions and red being most attractive.

All-atom simulations are unable to reach the time scales needed to study phase separation with current state-of-the-art computational hardware resources and sampling methods. We therefore introduce a coarse-grained representation of the protein, in which each residue is represented as a single particle (Fig 1a). The model takes into account the chemical properties of the 20 naturally occurring amino acids, listed in S1 Table, thus making it sequence specific. The potential energy function contains bonded, electrostatic, and short-range pairwise interaction terms. Bonded interactions are modelled by a harmonic potential with a spring constant of 10 kJ/Å^2^ and a bond length of 3.8 Å. Electrostatic interactions are modeled using a Coulombic term with Debye-Hückel [50] electrostatic screening to account for salt concentration, having the functional form:

$$\begin{array}{c} E_{ij}\left(r\right)=\frac{q_{i}q_{j}}{4\pi Dr}\text{exp}\left(-r/\kappa \right), \end{array}$$ (1)

in which *κ* is the Debye screening length and *D* = 80, the dielectric constant of the solvent medium (water). For all the simulations for which phase diagrams are generated, a Debye screening length of 1 nm, corresponding to an ionic strength of approximately 100 mM, is used. When determining *R*_*g*_ for IDPs from the literature, ionic strength is set to match that from the experimental results, as listed in (S2 Table). The short-range pairwise potential accounts for both protein-protein and protein-solvent interactions. Here we have introduced two different models: the first is based on amino acid hydrophobicity [51, 52] and uses a functional form introduced by Ashbaugh and Hatch [53]; the second is based on the Miyazawa-Jerningan potential [54] with the parameterized functional form taken from Kim and Hummer [55].

#### Hydrophobicity scale (HPS) model

The first model uses a hydrophobicity scale from the literature [52] to describe the effective interactions between amino acids. For use in the coarse-grained model, the atomic scale is first summed up to obtain a residue scale and is then scaled to the range from 0 to 1. The hydrophobicity values, *λ*, used for the 20 amino acids can be found in S1 Table and S1 Data. The arithmetic average is set as the combination rule for both the pair interactions *λ* between two amino acids and the size *σ* (S2 Data), of the amino acids (i.e., hydrophobicity scale *λ*_*i*,*j*_ = (*λ*_*i*_+*λ*_*j*_)/2 and amino acid size *σ*_*i*,*j*_ = (*σ*_*i*_+*σ*_*j*_)/2). The combined pairwise interaction strengths for each amino acid pair are shown in Fig 1e. The Ashbaugh-Hatch functional form [53] which has previously been applied to the study of disordered proteins [56], allows the attractiveness of the interactions to be scaled by *λ* (Fig 1b), and is described by,

$$\begin{array}{c} \Phi \left(r\right)=\{\begin{array}{cc} \Phi_{LJ}+\left(1-\lambda \right)\epsilon , & \text{if}\;r\leq 2^{1/6}\sigma \\ \lambda \Phi_{LJ}, & \text{otherwise} \end{array} \end{array}$$ (2)

in which Φ_*LJ*_ is the standard Lennard-Jones potential

$$\begin{array}{c} \Phi_{LJ}=4\epsilon [{\left(\frac{\sigma }{r}\right)}^{12}-{\left(\frac{\sigma }{r}\right)}^{6}]. \end{array}$$ (3)

The pair potential for the least hydrophobic amino acid at a *λ* value of 0 consists of only the repulsive term, making it equivalent to the Weeks-Chandler-Andersen functional form [57]. The model contains one free parameter *ϵ*, which determines the absolute energy scale of the short-ranged interactions and is set to be constant across all pairs. To determine the optimal *ϵ*, *R*_*g*_ was calculated for a set of IDPs (S2 Table) using our model, and compared with available experimental *R*_*g*_ data. Obtaining accurate estimates of *R*_*g*_ from FRET and SAXS experimental data requires some care, as has recently been noted [58–61]. Since FRET probes an intramolecular pair distance, inferring *R*_*g*_ requires the assumption of an underlying polymer model with known pair distance distribution and related *R*_*g*_. It has been shown that the commonly used Gaussian chain model works reasonably well for IDPs in the absence of chemical denaturants, but it breaks down when such denaturants are added [58, 62]. This is because the polymer scaling exponent *ν* ≈ 1/2 for IDPs without denaturants present, so that the Gaussian chain is a reasonable approximation for the denaturant-free conditions we are concerned with. We obtain the *R*_*g*_ using a Gaussian chain model with a dye correction of 9 residues, as previously described [59, 63]. For SAXS, Guinier analysis is challenging because the approximation is only valid for a small range of *q* where the data tends to be noisy; when fitting a larger range of scatter angles, it tends to underestimate the *R*_*g*_ [58]. A proper treatment of SAXS data requires a model that can also fit data at wider angles [58–60, 64]. Despite the limitations of the presently used data set, we expect that the systematic errors introduced by data analysis methods are still substantially smaller than the the deviation of the fit from experiment. However, a finer optimization of the model may require both the FRET and SAXS experimental data to be more accurately analyzed.

**Fig 2:**
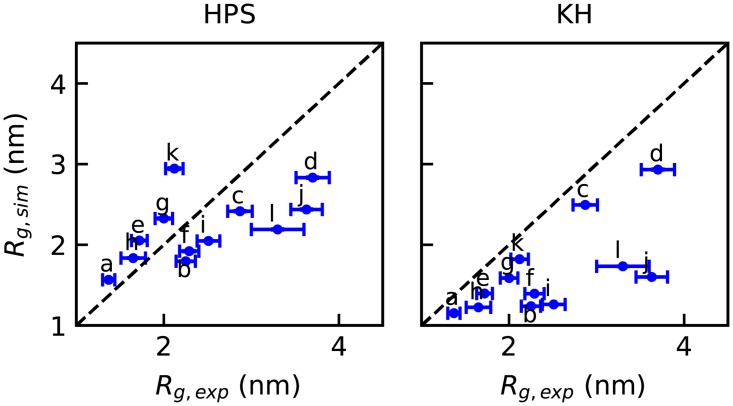
Parameterization of coarse-grained models: Comparison between radius of gyration of various intrinsically disordered proteins from experiment, and from simulation with the optimal parameters.

**Fig 3:**
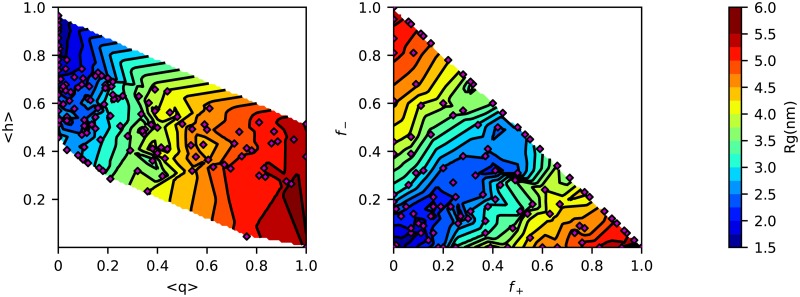
Randomly generated sequences of 100 amino acids follow the general trends expected from an Uversky type plot (left) and Pappu type plot (right). Axes are: mean hydrophobicity per residue 〈*h*〉, mean net charge per residue, 〈*q*〉 and fractions of positively *f*_+_ and negatively *f*_−_ charged residues. For both plots, the color represents average *R*_*g*_, and contour lines are spaced every 0.25 nm. The location of each tested sequence is represented by a purple diamond.

Fig 2 and S1 Fig show that an *ϵ* of 0.2 gives the greatest similarity to the experimental size of these unfolded proteins. In order to test if the model can capture the degree of collapse for folded and disordered sequences, we generated 131 sequences of 100 amino acids with properties covering a wide range of net charge and hydrophobicity values, and determined *R*_*g*_ from simulation. In Fig 3, we present the *R*_*g*_ of these sequences in a Uversky type plot [65] and in a Pappu type plot [66], both of which have been widely used to characterize sequence properties of proteins. *R*_*g*_ values are observed ranging from 1.5 to 6.0 nm, and the predictions are good for naturally occurring test sequences. The larger *R*_*g*_ values obtained for some of the synthetic sequences are outside the range observed for natural sequences in Fig 2, however this is because the extreme synthetic sequences are essentially polyelectrolytes which are rare in nature. Although we do not have experimental data for such sequences, we note that the model still makes accurate predictions for the most charged protein in our data set, Prothymosin *α*-N (S2c Table), which has a net charge of -43 (-0.384 per residue), mean hydrophobicity of 0.555, and *R*_*g*_ of 2.87 nm. It is clear that the HPS model describes the known sequence-specific features of the disordered proteins, that is, a small mean hydrophobicity scale and a large mean net charge. The Uversky plot in Fig 3 shows a correlation of *R*_*g*_ with both hydrophobicity and mean charge per residue as seen in experiment [63]. It does appear that the correlation is stronger with net charge, while both factors were correlated with scaling exponents in earlier work [63]. This is partly because our sampled sequences span a larger range of charge, and also because charge and hydrophobicity are correlated in naturally occurring sequences, making it harder to separate their respective contributions. Even so, the correlation with charge does appear to be better in experiment [63].

#### Kim-Hummer (KH) model

A different model for short-range interactions has been previously developed and parameterized by Kim and Hummer to describe protein-protein interactions, using a variety of experimental data including the osmotic second virial coefficient of lysozyme and the binding affinity of the ubiquitin–CUE complex [55]. The potential function they used can be expressed in terms of Ashbaugh-Hatch potential function (Eqs 2 and 3), where

$$\begin{array}{c} \epsilon =|\alpha (\epsilon_{MJ}-\epsilon_{0})|, \end{array}$$ (4)

and

$$\begin{array}{c} \lambda =\{\begin{array}{cc} 1, & \text{if}\;\epsilon_{MJ}\leq \epsilon_{0}\\ -1, & \text{otherwise} \end{array} \end{array}$$ (5)

*ϵ*_*MJ*_ is from the Miyazawa-Jerningan statistical contact potential [54]. Regarding the choice of *α* and *ϵ*_0_, the original literature identifies six sets of parameters, differing in the treatment of interactions involving buried residues. Here we employ parameter set D (*α* = 0.228 and *ϵ*_0_ = −1.00 kcal/mol, S3 Table, S3 Data) for IDR, which generates a reasonable estimate of *R*_*g*_ for a list of IDPs (Fig 2), and parameter set A (*α* = 0.159 and *ϵ*_0_ = −1.36 kcal/mol, S4 Table, S4 Data) for the helicase domain, which was parameterized for interactions between folded proteins [55], The correlation between the parameters of the HPS and KH models for IDR is shown in Fig 1d. We repeat the analysis previously done with the HPS model on the same set of 100-mers (S2 Fig) to provide additional insight into how the two models compare with regard to relative interaction strength of hydrophobic and electrostatic interactions. Both attractive and repulsive forces are stronger in the KH model than in HPS, thus there is a stronger dependence of *R*_*g*_ on hydrophobicity, especially for sequences with low charge.

### Simulation framework

#### Slab method

**Fig 4:**
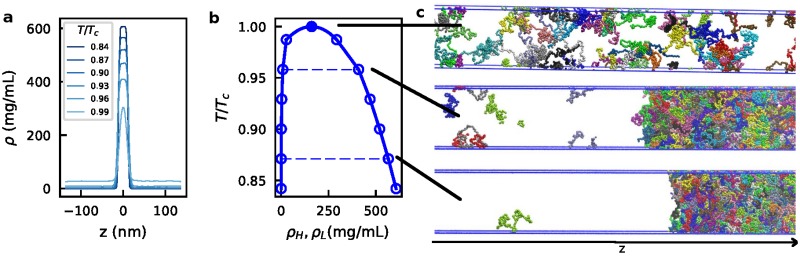
Slab sampling method and typical configurations in different regions of the phase diagram for FUS WT. a) Slab density profile of FUS WT at a set of temperatures where two phases coexist. The raw density plot is highly symmetrical, suggesting good convergence of simulations. b) Phase diagram of FUS WT obtained from the density profile. c) Typical configurations of FUS slab simulations at different temperatures.

In order to determine the phase diagram of the disordered proteins, we utilize a method [46, 67], in which the high-density (concentrated) phase, with surfaces normal to *z*, is simulated in equilibrium with the low-density (dilute) phase as visualized in Fig 4c, S1 and S2 Movies. This allows the determination of the equilibrium density (or concentration) of proteins in each phase and consequently, the critical temperature, as described in more detail below. This initial equilibration is conducted for 100 ns in the NPT ensemble, starting from a dispersed phase of protein chains with periodic boundary conditions at 150 K, maintained by a Langevin thermostat with a friction coefficient of 1 ps^−1^, and 1 bar, maintained by a Parrinello-Rahman barostat [68]. A time step of 10 fs is used for all the simulations. The box size is first scaled to roughly 15 nm (25 nm for full length LAF-1) for both *x* and *y* axes and then equilibrated along the *z*-axis using anisotropic pressure coupling. Depending on the protein of interest and the pairwise potential, the length of the *z*-axis can vary. The *x*- and *y*- dimensions were set to 15 nm which is sufficient to prevent to most of the chains (>99% estimated by a random-coil model for a 170-residue chain) from interacting with their periodic images. Then the *z*-dimension of the box was extended to 280 nm (∼20 times larger than the initial z-dimension box size). Simulations are then conducted at multiple temperatures for ∼5 *μ*s using constant temperature and volume with a Langevin thermostat with a friction coefficient of 0.01 ps^-1^. The temperature is gradually increased from 150 K to the targeted temperature over the first 100 ns. The next 1 *μ*s of simulation is discarded as equilibration, and the remainder (at least 4 *μ*s) is used for further analysis. Simulations were conducted using the LAMMPS [69] and HOOMD-Blue v2.1.5 [70] software packages in order to benefit from both CPU and GPU resources.

We took several measures to verify that the initial configuration, system size and number of steps are sufficient to obtain well-converged thermodynamic properties of the system. First, we find that a simulation starting from a fully dispersed configuration, in which chains are put far from each other, but having the same periodic box geometry, will eventually coalesce to form a concentrated phase and generate a similar density profile (after 4 *μ*s) to a simulation starting from a slab-like initial configuration (S3 Fig). Therefore a slab-like initial configuration reduces the length of the simulation required for convergence. Second, we do not see a quantitative difference of the results between the two halves of a 10 *μ*s simulation (S4 Fig), suggesting 5 *μ*s is sufficient for convergence of the system. Third, we have also found that a system with 100 chains of 163 residue FUS is sufficiently large to avoid finite-size effects, as the results are identical to those from a similar set of simulations containing 200 chains (S5 Fig).

#### Slab density profile

To determine the density profile along *z*, we first center the trajectory on the slab for each frame. The slab is defined as the cluster with the largest number of chains. Clustering was done according to center-of-mass distance between chain pairs, where chains with center-of-mass distances less than 5 nm are considered to be in the same cluster except for full length LAF-1, with which we use a cutoff of 7 nm due to its larger size. The density profile along *z* is then generated as shown in Fig 4a and S6a Fig. If phase separation occurs, we obtain the protein concentration of the dilute and concentrated phases (*ρ*_*L*_ or *ρ*_*H*_) by using the average concentrations when |*z*| >50 nm or |*z*| <5 nm respectively. Protein concentration is reported in units of mg/mL.

#### Phase diagram

The critical temperature *T*_*c*_ can be obtained by fitting

$$\begin{array}{c} \rho_{H}-\rho_{L}=A{\left(T_{c}-T\right)}^{\beta } \end{array}$$ (6)

where *β* is the critical exponent which is set to 0.325 (universality class of 3D Ising model [71]) and *A* is a protein-specific fitting parameter. For fitting to this equation, a specific range of temperatures must be used. The minimum fitting temperature, *T*_1_, is chosen as the lowest temperature where *ρ*_*L*_ is nonzero. The maximum fitting temperature *T*_2_ must be below the critical temperature as Eq 6 can only describe the behavior below *T*_*c*_ (S6c Fig). To determine the optimal value for *T*_2_ we calculate the relative error of *T* when fitting *T* as a function of *ρ*_*H*_ − *ρ*_*L*_ using different test values of *T*_2_. This error will be large if *T*_2_ is greater than *T*_*c*_ (S6d Fig). We can then obtain a typical phase diagram as shown in Fig 4b and S6b Fig, in which the *ρ*_*L*_ and *ρ*_*H*_ when *T* < *T*_*c*_ are determined from averaging different regions of the slab density profile as described above, and *T*_*c*_ and the corresponding *ρ*_*c*_ are from fitting Eq 6 (S6c Fig). Fig 4c shows visualizations of the different states of coexistence captured by these simulations. When the system is above *T*_*c*_, the slab evaporates to a supercritical protein solution. When the temperature is below *T*_*c*_, we see coexistence of two phases: one phase with free monomers and the other with many proteins in a condensed, liquid-like assembly. The number of free monomers decreases with decreasing temperatures to concentrations comparable with protein concentration in the dilute phase observed by experiment [6, 25]. The critical temperatures for all sequences presented in this work are listed in S5 Table.

We have also fit our simulated phase diagram with Flory-Huggins theory [72, 73] by using Eq. S11 of reference [74]. There are three fitting parameters used in the original literature [74]: *A* and *B* are the temperature-independent and dependent terms in the interaction strength *χ* whereas *ρ* is the protein density. We expect that in our coarse-grained simulation, the entropic contribution to *χ* will be negligible. Indeed, we find that if we allow three fitting parameters, *A* is usually one or two orders of magnitude smaller than *B*/*T*. In order to improve the robustness of the fitting, we therefore set *A* to be zero and only use two free parameters, *B* and *ρ*. We list *ρ* and *χ* calculated from *B* of each sequence in S6 Table.

#### Simulations with folded domain

Proteins which undergo LLPS usually contain multiple domains, including both folded and disordered domains [75]. Recently, Riback et al. found that poly(A)-binding protein Pab1 exhibits LLPS behavior in the absence of its disordered domain, but does not in the absence of the folded domains [14], contrary to the notion that intrinsic disorder is necessary for phase separation. Since both intrinsically disordered and folded domains can form favorable intermolecular interactions stabilizing the high density phase, it is only natural that they may both contribute to the LLPS behavior, and the contributions may be different from protein to protein. We use full length LAF-1 which contains a folded domain and two disordered domains, as a test case to see how the proposed framework will accommodate folded proteins.

The structure of the folded domain (helicase) of LAF-1 has not yet been solved, so we have used homology modelling and the Modeller v9.17 package [76] to embed the LAF-1 helicase sequence into its homologue with a solved crystal structure, VASA [77] (S7 Fig).

Here we employ the KH model with parameter set A (*α* = 0.159 and *ϵ*_0_ = −1.36 kcal/mol) for all interactions involving the helicase domain, and parameter set D for disordered-disordered interactions as before. The reason for this is that a 12-6 potential allows buried residues to make a significant contribution to binding energies of folded domains, which will have a stronger effect on the affinity than the specificity of the interactions. Model *A* was parameterized including such interactions for folded proteins, and is therefore appropriate for use in our model in describing interactions involving folded proteins. Model *D* was parameterized using a screening term to reduce the effect of buried residues, and is therefore appropriate for describing interactions between disordered regions where all residues are essentially fully exposed. A universal set of parameters would require a different functional form.

When the structure of the folded domain is modelled, we treat the helicase as a rigid body (i.e., “fix rigid” command in LAMMPS or “md.constrain.rigid” command in HOOMD-Blue) in the simulation so that the structure of the folded domain is preserved. Interactions between residues within the same rigid body are neglected. The mass of the rigid body is scaled to be 0.5% of the original mass in order to accelerate rigid body dynamics. When calculating the density of the folded domain, the mass is scaled back to match the mass of the original folded domain with all residues. The folded domain can in principle also be simulated using harmonic restraints instead of rigid constraints, which would allow additional flexibility. However there is a clear advantage for using rigid body dynamics in terms of computational efficiency.

## Results

### Phase separation of FUS and its phosphomimetic mutants

As a first application of our model to LLPS, we use the prion-like LC domain of the protein FUS (FUS-LC) which is sufficient to induce LLPS in vitro in the absence of other biomolecules [15]. FUS-LC is an ideal system to test our model as it is fully disordered and displays very low secondary structure content [6]. The sequence is largely uncharged, with only 2 anionic aspartate residues within its 163 amino acid sequence. To test for sequence-specific effects, we conducted simulations for several different variants of the FUS-LC peptide, wild-type and four phosphomimetic mutants where a set of the 12 naturally phosphorylated threonine or serine residues are mutated to glutamate. [78] The first of these mutants is the 12E mutant, which contains all 12 glutamate substitutions, and does not undergo LLPS under similar conditions to FUS WT [79]. We additionally test the 6E mutant reported in the same work [79], and two designed variations of the 6E mutant, termed 6E’ and 6E* which maximize and minimize, respectively, the clustering of charged residues within the sequence under the constraints of preserving the amino acid composition of 6E, and only mutating naturally occurring phosphorylation sites. [80]

Utilizing the slab method, we determine the range of temperatures at which the simulated FUS chains separate into two phases, and calculate the coexistence curve using both the HPS and KH models. The concentration of the dilute phase gives the predicted critical/saturation concentration of the protein, the concentration above which it will begin to form droplets in solution. The concentration of the dilute phase is on the order of 0.1-10 mg/mL over the tested temperature range, consistent with typical concentrations used to observe phase separation of FUS WT in vitro [6] (∼1-5 mg/mL). We find that the critical temperature differs between the two models for FUS WT. However the coexistence curves and the phase diagrams are qualitatively similar (S8a Fig), as are the intermolecular contact maps (S9 Fig).

**Fig 5:**
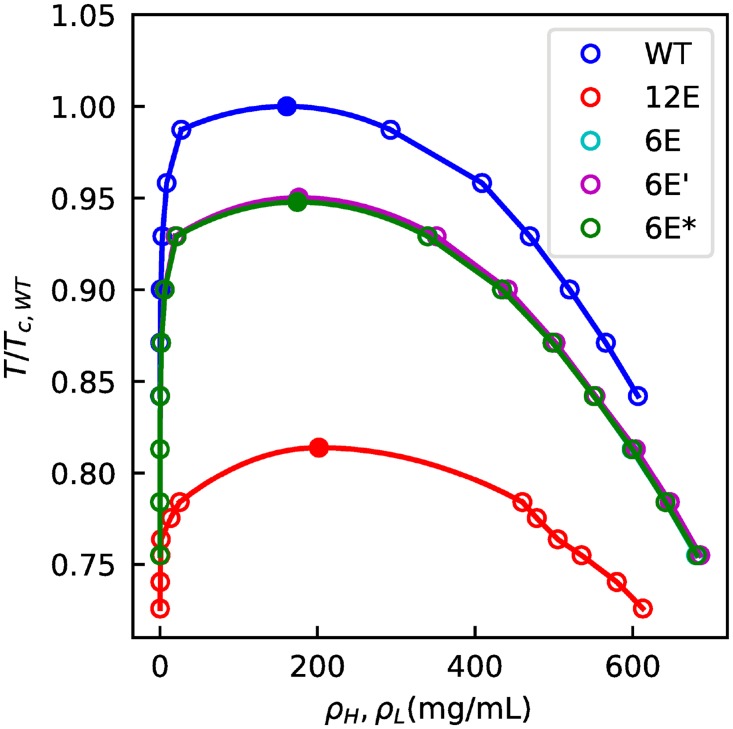
Phase diagram for FUS WT, 6E variants and 12E. Temperatures are scaled by the critical temperature of FUS WT.

To evaluate the impact of the phosphomimetic mutations, we determine the phase diagram for FUS WT, 6E, 6E’, 6E*, and 12E using the HPS model (Fig 5). The 12E mutant phase separates at a much lower temperature, with the critical temperature smaller than even the lowest temperature at which we can observe coexistence between two phases for FUS WT (due to the prohibitively small concentration of the low-density phase). This is consistent with the experimental observation that FUS 12E does not phase separate in contrast to FUS WT at similar conditions [79]. The 6E mutants all lie between the two extreme cases, and have nearly identical phase diagrams. While the difference of just 6 amino acids results in a greatly altered phase-separation ability from wild type, the rearrangement of these mutations does not have such an effect. However, these mutations were done under very strict constraints which do not allow for much change in the degree of charge clustering. We also calculate the inter-chain contacts, defined as two amino acids of different chains within 2^1/6^ *σ*_*ij*_ of each other. There are no specific contacts formed in either of the cases (S9 Fig), suggesting that LLPS of FUS WT is not driven by a specific region within the protein sequence. However, when comparing the different 6E mutants at the same temperature, the degree to which different regions of the peptide interact are greatly affected (S10 Fig). This shows that despite having nearly identical phase diagrams, the interactions involved in phase separation can vary. The average interaction strength per residue *χ* can also be obtained by fitting the phase diagram to Flory-Huggins theory [72, 73] as shown in S11 Fig. We find that there is a clear decreasing trend of *χ* from 0.410 to 0.325 kcal/mol with increasing number of phosphomimetic mutations (S6 Table).

To further check the liquid-like nature of the concentrated phase, we calculate the mean squared displacement (MSD) as a function of time using NVT simulations for WT, 6E and 12E at 500mg/mL (S12 Fig, S3 Movie). For each, there is a linear region with non-zero slope suggesting that the concentrated phase is liquid-like, and not a solid aggregate. The diffusion coefficient from fitting the linear region is ∼3x10^−6^ cm^2^/s, three orders of magnitude larger than measured in the experiment (4x10^−9^ cm^2^/s [6]) as can be expected from a coarse-grained simulation and as we are using low friction Langevin dynamics. Finally, we check monomer radius of gyration in both the dilute and concentrated phase and find that chains in the concentrated phase are generally more extended than those in the dilute phase (S13 Fig).

### Phase separation of IDR and full length LAF-1

Next, we apply our model to DEAD-box helicase protein, LAF-1, which has been shown to phase separate as both its IDR and as full length, including a 437-residue folded domain, in vitro [25]. To test the effect of inclusion of folded domains, three variants of LAF-1 sequences have been simulated, including the N-terminal IDR of LAF-1, the helicase domain, and full length LAF-1 with both the IDR and folded domain as well as the prion-like C-terminal domain which is also disordered. The IDR sequence is of similar length to FUS, but contains a larger fraction of charged amino acids, (∼26%) compared to FUS WT (∼1%), and FUS 12E (∼9%), and includes both attractive and repulsive electrostatic interactions. For LAF-1 IDR, we simulated the phase diagram with both KH and HPS models. As was the case for FUS WT, the phase diagrams are qualitatively similar between the two models (S8b Fig).

**Fig 6:**
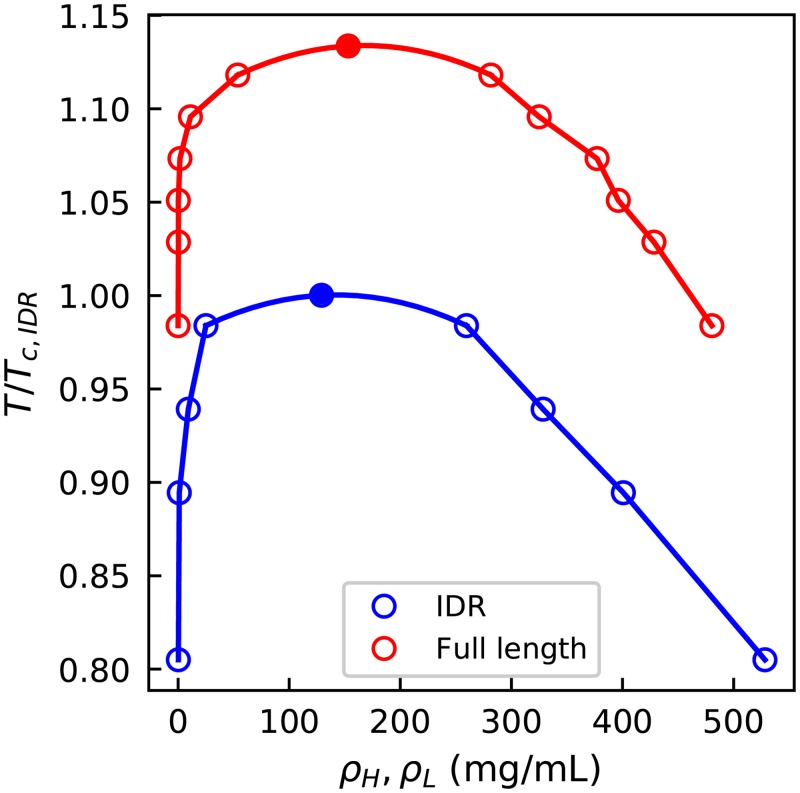
Phase diagram of IDR (blue) and full length (red) LAF-1. Temperatures are scaled by the critical temperature of IDR LAF-1. The corresponding slab density profiles are provided in S15 Fig.

In Fig 6 we compare the phase diagrams of the full length and IDR regions of LAF-1. The phase diagram for the full length protein is shifted toward higher temperatures, and suggests a smaller saturation concentration as compared to the LAF-1 IDR alone at the same temperature. The results for the helicase domain alone also clearly show phase separation (S14 Fig). The experimental phase boundary in ∼120 mM NaCl is ∼0.05 mg/mL for full length LAF-1, but ∼0.4 mg/mL for the isolated IDR [25]. Even though we cannot accurately estimate the low protein concentrations in the dilute phase so as to quantitatively compare with the experimental values, we do see an increase in the saturation concentration when adding the folded domain as has been seen by experiment. We note that the concentrations obtained from the high density phase are much higher than recently estimated by Wei et al. [81], however, they are quite comparable with those measured by Brady et al. for the similar DEAD-Box Helicase protein Ddx4 [74]. Fitting the phase diagram to Flory-Huggins theory, we obtain the average interaction strength per residue, *χ*, of LAF-1 IDR (S8 Fig). The *χ* is 0.270 kcal/mol for KH model and is 0.298 kcal/mol for HPS model, both comparable to 0.3 kcal/mol obtained from experimental Ddx4 data [74].

The reason for the change of critical temperature upon inclusion of the folded domain is likely two-fold. First, the folded domain contains more hydrophobic residues with an average hydrophobicity of 0.664 (0.579 for the surface residues) in contrast to 0.520 for LAF-1 IDR (S1 Table), therefore strengthening the intermolecular attraction. In addition, providing more interaction sites per chain generally favors a higher critical temperature, because more interactions can be formed with a smaller loss of entropy, an effect commonly referred to as multivalency [35]. The impact of multivalency on the phase coexistence will be investigated explicitly in the next section.

**Fig 7:**
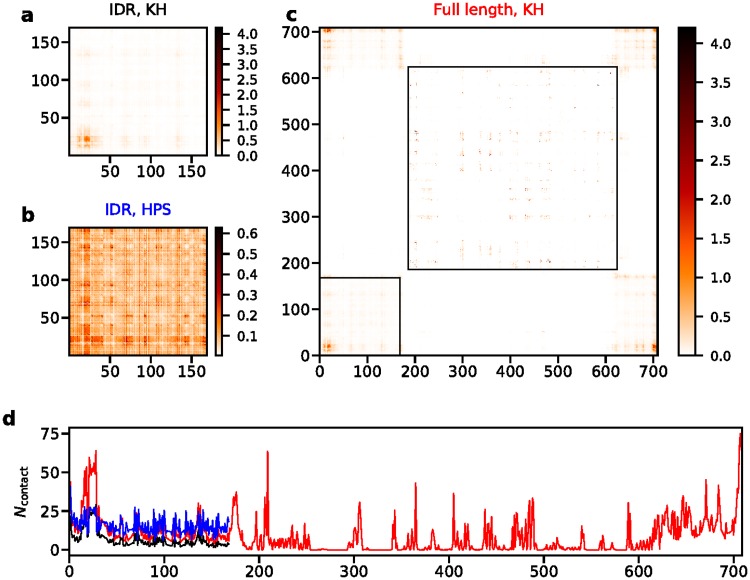
Number of intermolecular contacts per frame for LAF-1 with different models at 220K. a) Contact map of IDR LAF-1 with KH model. b) Contact map of IDR LAF-1 with HPS model. c) Contact map of full length LAF-1 with KH model. Black boxes illustrate the N-terminal IDR and the folded domain. d) Number of intermolecular contacts per residue per frame for IDR LAF-1 with KH model (black), IDR LAF-1 with HPS model (blue) and full length LAF-1 with KH model (red).

In the concentrated phase, we also investigate the intermolecular contacts in Fig 7. Unlike the case of FUS, there are regions along the sequence where there is a relatively high propensity to form contacts, (residue 21 to 28, RYVPPHLR) and (residue 13 to 18, NAALNR). These regions are present in both the IDR with the KH and HPS model (Fig 7a and 7b) and in the full length protein (Fig 7c). The central region of these two segments is composed of uncharged amino acids, suggesting the importance of hydrophobic patches in the sequence even with a large fraction of charged residues. As is shown in both 1D and 2D contact maps (Fig 7a, 7c and 7d), the pattern, and number of contacts within the IDR look similar in both the IDR and the full length LAF-1 simulations. This observation also applies to the helicase domain in the helicase only, and full length LAF-1 simulations (S16 Fig). This suggests that the key residues contributing to the droplet formation are the same for the disordered peptide with and without the folded domain (Fig 7d). Additionally, the disordered part of the protein (including both the N-terminal and C-terminal disordered regions) contributes more contacts than the folded domain in the simulation of full length LAF-1, consistent with the experimental observations that the disordered region of LAF-1 is the driving force for the LLPS [25]. The intramolecular contact map in the two phases (S17 Fig) supports the change of *R*_*g*_ (S13 Fig) in that the peptide has fewer long range contacts in the concentrated phase than in the dilute phase.

We additionally calculate the mean squared displacement (MSD) as a function of time for all the three variants of LAF-1 (i.e., IDR, helicase and full length) using NVT simulations at concentrations predicted for the condensed phase at 210K (S4, S5 and S6 Movies), to see how the different regions affect the diffusion of the protein within the concentrated phase. There is a linear region with non-zero slope for all the variants (S12 Fig) suggesting liquid-like behavior. The IDR has a much larger diffusion coefficient than both the full length and the helicase domain of LAF-1 making it the most mobile of the three. This is likely due to its flexibility as well as the lower concentration. The diffusion coefficient for full length LAF-1 is an order of magnitude larger than that of just the helicase domain, further supporting the importance of the flexible region for maintaining liquid-like behavior of proteins inside the droplet.

### Multivalency of IDRs

Multivalency has shown to be important in driving LLPS in experimental studies [20, 35] where proteins with a higher number of repeated units begin to form droplets at lower concentrations. Usually multivalency is used to describe a certain number of specific interaction sites per molecule. For polymers, there is inherently a large number of possible interactions between molecules, so for well-mixed sequences specific residue-residue interactions are less likely to play a role in assembly. Nonetheless, increasing the chain length will (for a given sequence composition) increase the number of available interaction sites per chain, and thus, increase multivalency of the system.

**Fig 8:**
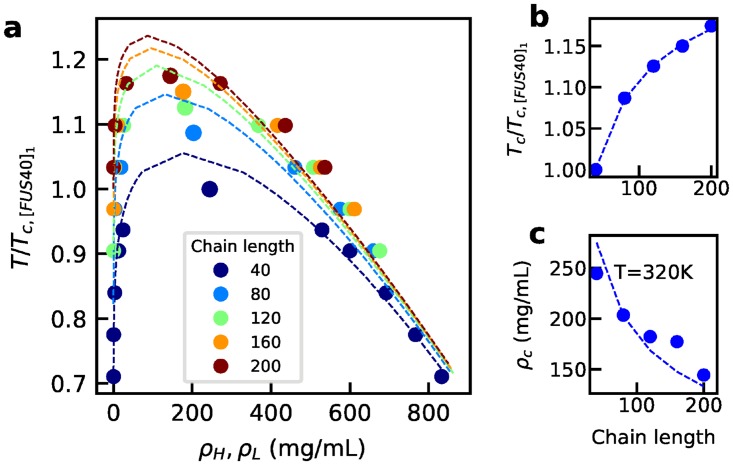
Phase separation of truncated FUS fragments of different lengths. a) Phase diagram for each peptide. Dashed lines show the fitting to binodal of Flory-Huggins theory. b) The critical temperature. Dashed lines show the fitting using relation $T_{c}\propto N/{\left(\sqrt{N}+1\right)}^{2}$ with prefactor as the fitting parameter. c) The critical concentration. Dashed lines show the fitting using relation $\rho_{c}\propto 1/\left(\sqrt{N}+1\right)$ with prefactor as the fitting parameter. Temperatures are scaled by the critical temperature of [FUS40]_1_. The corresponding slab density profiles are provided in S18 Fig.

In order to investigate the mechanism of such behavior, we use a model system where we take the first 40 residues from FUS LC and make several repeated units in the form of [FUS40]_*n*_, in which n = 1, 2, 3, 4 and 5. We then conduct multiple slab simulations for each of these sequences, keeping the total number of atoms constant (see detailed system size in S6 Table). The phase diagrams of [FUS40]_*n*_ in Fig 8a show that the phase boundary shifts to higher temperatures and lower concentrations with increasing chain length.

To understand the mechanism of such dependence, we apply Flory-Huggins theory [72, 73], which has previously been used to understand IDP phase separation [9, 37, 38, 74], to fit the phase transition properties obtained by molecular dynamics simulations when varying the chain length *N*. If we assume that each solvent molecule occupies one lattice position, we can fit all five phase diagrams from different chain lengths to the binodal of Flory-Huggins theory using the same average interaction strength per residue *χ* and protein density *ρ* (Fig 8a and S6 Table). Since there is analytic solution for the critical temperature and concentration from Flory-Huggins theory: the critical temperature $T_{c}\propto N/{\left(\sqrt{N}+1\right)}^{2}$ and the critical concentration $\rho_{c}\propto 1/\left(\sqrt{N}+1\right)$, we can also fit our simulated *T*_*c*_ and *ρ*_*c*_ as a function of the chain length with these approximate equations (assuming prefactor as the fitting parameter), as shown in Fig 8b and 8c. These results suggest that the phase diagram dependence on the chain length can be described by Flory-Huggins theory. The term that is sensitive to changes in chain length is the mixing entropy per segment. With increasing the chain length, the mixing entropy per segment decreases, and therefore the critical temperature increases. It would then be easier to observe LLPS with a longer chain at the same temperature, in the sense that the dilute-phase concentration is smaller, consistent with experimental observations [20].

This factor should be considered when making mutations to protein sequences with the aim of understanding the molecular origin of LLPS: in general, chain truncation or extension will disfavor or favor LLPS, respectively, regardless of the sequence-specific effects. Similarly, when cutting a larger protein into fragments in order to evaluate the contribution of each to driving LLPS, it is expected in general that longer fragments will be able to phase separate at a higher temperature.

## Discussion

We have introduced a general framework for conducting molecular dynamics simulations of LLPS leading to protein assemblies constituting many membraneless organelles. Coarse-graining to amino-acid-resolution gives access to length and time scales needed to observe this phenomenon, and to achieve convergence of thermodynamic observables (i.e., phase diagram, critical temperature and protein concentration in the dilute and concentrated phases) while preserving sequence-level information, thus allowing observance of changes induced by mutations to the protein sequence. The force fields utilized in this work are based on previously determined, knowledge-based potentials, parameterized to accurately represent the radius of gyration of disordered proteins, but the framework is also flexible to incorporate other residue-based pairwise interaction potentials. The two force fields generate similar intermolecular contact maps within the concentrated phase, suggesting that description of the weak nonspecific interactions in IDPs can be captured easily with different models.

We have tested the framework and the two force fields with two model systems, which undergo phase separation in vitro, yielding phase diagrams, thus giving the critical temperature, and saturation concentration at the tested temperatures. Despite the simplicity of the currently used potentials, and the fact that they were exclusively optimized based on the properties of monomeric proteins, we demonstrate the ability to predict how various perturbations to the system can change the LLPS. In the case of FUS LC, the model is able to capture the experimentally observed variation of phase diagram when introducing mutations. In LAF-1, the model is able to capture the experimentally observed difference between the phase separation of full length and truncated disordered-only sequences. We also show that the inclusion of the disordered parts function to increase the diffusion of LAF-1 within the condensed phase.

We have also investigated an important feature of LLPS regarding the dependence of phase behavior on chain length, which is well established in polymer physics and was previously observed in experiment [20]. We show that there is an upward shift in the phase diagram (temperature-concentration) with increasing chain length. At a given temperature, the saturation concentration will be higher for shorter chain lengths. Both the critical temperature and concentration are in good agreement with Flory-Huggins theory and therefore suggest the behavior can be explained by relative loss of entropy. With this in mind, if the phase behavior of a protein of interest cannot be observed in vitro, making repeated units might be a convenient way to shift the phase diagram enough that LLPS will be observable under more reasonable experimental conditions. One must also consider this effect when making changes to protein length, such as His tags, or cleavage of a certain section of residues, and how just the change in chain length may affect the coexistence.

Additionally, we measure certain important properties of proteins within the concentrated phase for the two model systems such as intermolecular contact propensities, which are quite difficult to resolve experimentally. With FUS LC, the intermolecular contacts are evenly distributed throughout the length of the peptide, suggesting that non-specific hydrophobic interactions are largely responsible for driving the phase-separation. For LAF-1, we observe enhanced intermolecular contacts within a specific region (residue 21-28), largely composed of hydrophobic amino acids, suggesting that even though LAF-1 contains 26% charged residues, hydrophobic interactions are still an important driving force for LLPS.

There are some features that cannot be captured in the presented model, but can be added in future work. First the absolute temperature of the simulation is not comparable to experiment. The phase behavior at the lower critical solution temperature, which is observed in some disordered peptides experimentally [31], cannot be captured, either. Both require the addition of a temperature dependent solvation energy term into the framework, and more comparison with experimental *R*_*g*_ data (or other relevant data). Second, we have not fully tested the ionic strength dependence of the current model because of the breakdown of Debye-Hückle electrostatic screening at high ionic strength, even though the trend of LAF-1 experiments when varying salt concentration is captured in the current model. However, we do not see any ionic strength dependence for FUS LC, which is inconsistent with experiment [6]. To capture salt dependence in proteins with negligible charged amino acid content, it may be necessary to include a description of “salting-out” effects, i.e., the change of solubility with salt concentration as captured by the Hofmeister series. In S19 Fig, we show that the literature-known amino acid specific salting-out coefficients [82–85] are strongly correlated with the hydrophobicity scale and therefore it may be possible to model the salting-out effect with an additional energy term using the same hydrophobicity scale. In the future, we would also like to introduce additional handles (such as a structure-based potential for intramolecular interactions) to allow for conformational changes within the folded parts of a chain. This will allow us to study LLPS of proteins with small populations of folded regions that are important for self-assembly.

## Supporting information

S1 Fig Comparison of *R*_*g*_ between simulations and experiments with different *ϵ* parameters for HPS model. (PDF)

S2 Fig Randomly generated 100-mers in a Uversky (left) and Pappu (right) plot to show the dependence of *R*_*g*_ on charge and hydrophobicity using the KH model. (PDF)

S3 Fig LAF-1 simulation started from dispersed state at 210K with KH-D model showing coalescence to a slab conformation after about 4 *μ*s. (PDF)

S4 Fig Comparison of density profiles between first 5*μ*s and last 5*μ*s of slab simulations of FUS WT. (PNG)

S5 Fig Comparison of FUS WT simulations with 100 (blue) and 200 (red) chains. (PDF)

S6 Fig Methodology used to determine the range of temperatures to fit Eq 6 in the main text. (PDF)

S7 Fig Homology modelling of helicase domain of LAF-1 using the structure of VASA. (PDF)

S8 Fig Comparison of the phase diagram generated with HPS (blue) and KH (red) model in FUS WT (left) and LAF-1 IDR (right). (PDF)

S9 Fig Inter- (upper) and intra-molecular (lower) contact maps for FUS WT at 260 K using HPS (left) and KH models (right). (PDF)

S10 Fig Intermolecular contacts for FUS 6E’ divided by that of FUS 6E* showing how the overall number of contacts forming within the slab changes between the two sequences. (PDF)

S11 Fig Phase diagram for FUS WT, 6E variants and 12E fitting to the Flory-Huggins theory. (PDF)

S12 Fig Mean squared displacement (MSD) as a function of time for FUS variants at 260K and 600 mg/mL (left), and LAF-1 at 210K and 260, 535 and 500 mg/mL for IDR, helicase, and full length respectively. (PDF)

S13 Fig Radii of gyration of the disordered proteins inside (blue) and out of (red) the slab. (PDF)

S14 Fig Phase diagram of IDR (blue), helicase (cyan) and full length (red) LAF-1. (PDF)

S15 Fig Slab density profiles of IDR (left), helicase (middle) or full length (right) LAF-1. (PDF)

S16 Fig Number of intermolecular contacts per frame for different LAF-1 variants at 220K. (PDF)

S17 Fig Number of intramolecular contacts per frame for LAF1 IDR with KH model at 200K. (PDF)

S18 Fig Slab density profiles of the repeated peptides of FUS fragment. (PDF)

S19 Fig The correlation between salting-out constant and hydrophobicity scale. (PDF)

S1 Table The amino acid parameters used in the HPS model. (PNG)

S2 Table List of intrinsically disordered or unfolded proteins with experimentally determined *R*_*g*_. (PNG)

S3 Table Interaction parameters (*ϵ*_*ij*_) used for KH-D model. (PNG)

S4 Table Interaction parameters (*ϵ*_*ij*_) used for KH-A model. (PNG)

S5 Table Summary of slab simulations and critical temperatures obtained. (PNG)

S6 Table List of parameters for fitting to Flory-Huggins theory. (PNG)

S1 Movie Slab simulation of FUS WT at 300K. (MP4)

S2 Movie Slab simulation of LAF-1 IDR at 250K. (MP4)

S3 Movie NVT simulation of FUS WT at 260K and 615.2 mg/mL. (MP4)

S4 Movie NVT simulation of LAF-1 IDR at 210K and 261.9 mg/mL. (MP4)

S5 Movie NVT simulation of LAF-1 Helicase domain at 210K and 535.5 mg/mL. (MP4)

S6 Movie NVT simulation of LAF-1 full length at 210K and 501.0 mg/mL. (MP4)

S1 Data Parameter file containing normalized hydrophobicity (*λ*) values for each amino acid. (TXT)

S2 Data Parameter file containing VDW radius (*σ*) used for each amino acid. (TXT)

S3 Data Parameter file containing KH model D pairwise energies (*ϵ*_*ij*_). (TXT)

S4 Data Parameter file containing KH model A pairwise energies (*ϵ*_*ij*_). (TXT)

S1 Document Document containing protein sequences, homology modelling and all supporting figures and tables. (PDF)
