# Supplementary figures and images for: Sequence determinants of protein phase behavior from a coarse-grained model
Source: PLoS Comput Biol. 2018 Jan 24;14(1):e1005941. doi: 10.1371/journal.pcbi.1005941 (PMC5798848; doi:10.1371/journal.pcbi.1005941)

$\varepsilon=0.10$   
 $\chi^2=77.4$

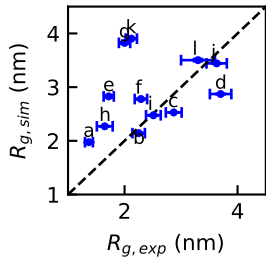

$\varepsilon=0.15$   
 $\chi^2=42.6$

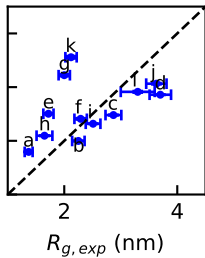

$\varepsilon=0.20$   
 $\chi^2=19.2$

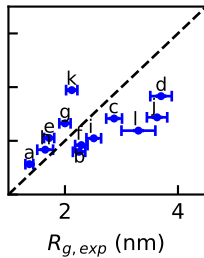

$\varepsilon=0.25$   
 $\chi^2=23.2$

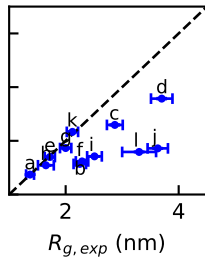

$\varepsilon=0.30$   
 $\chi^2=35.5$

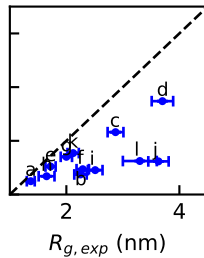

Supplement: S1 Fig — (PDF) [file pcbi.1005941.s001.pdf]

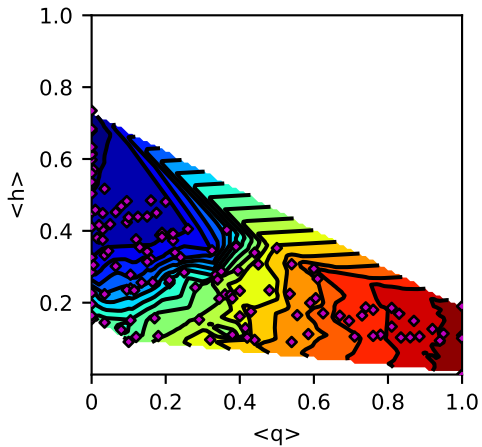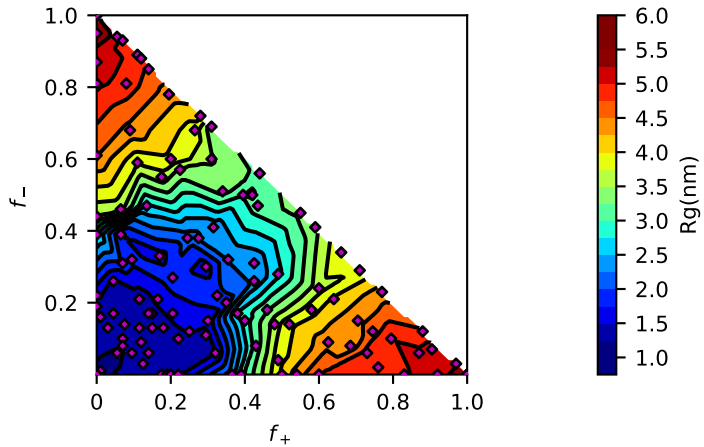

Supplement: S2 Fig — (PDF) [file pcbi.1005941.s002.pdf]

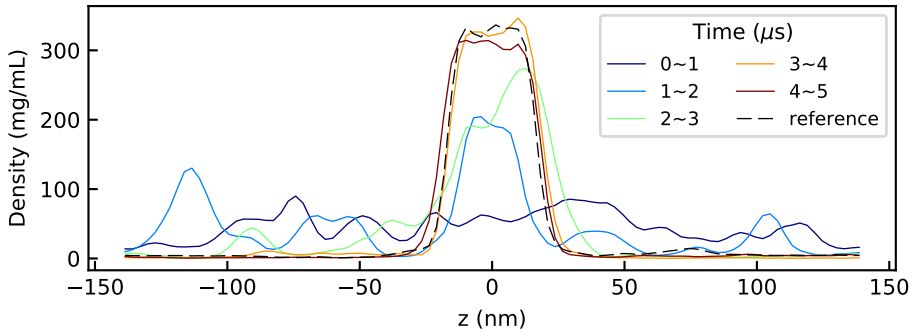

Supplement: S3 Fig — (PDF) [file pcbi.1005941.s003.pdf]

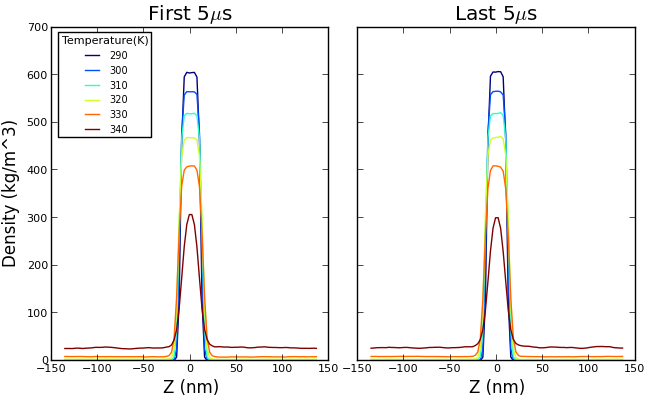

Supplement: S4 Fig — (PNG) [file pcbi.1005941.s004.png]

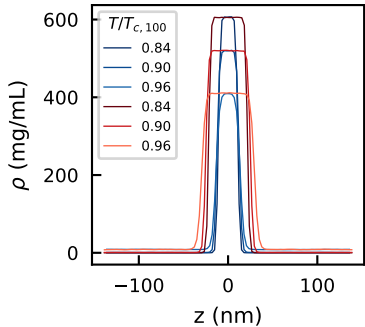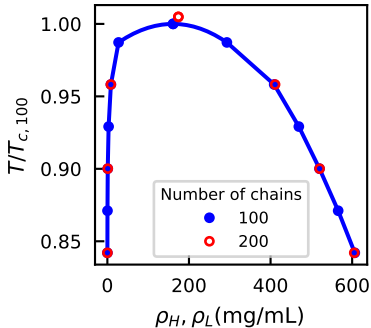

Supplement: S5 Fig — (PDF) [file pcbi.1005941.s005.pdf]

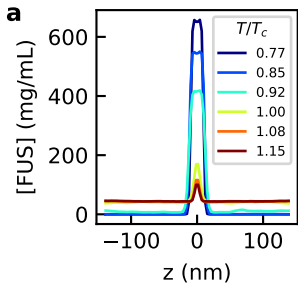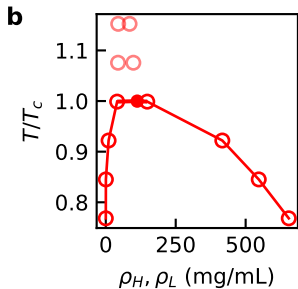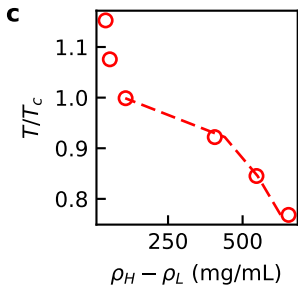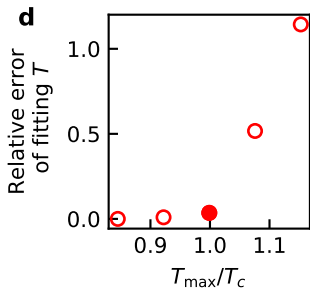

Supplement: S6 Fig — (PDF) [file pcbi.1005941.s006.pdf]

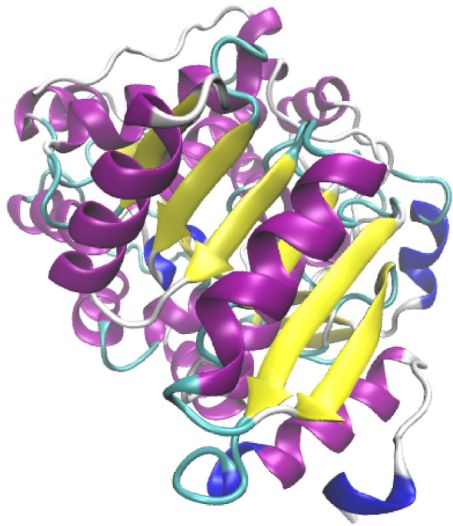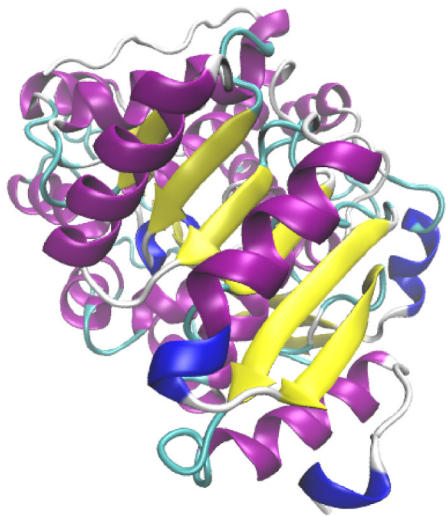

Supplement: S7 Fig — (PDF) [file pcbi.1005941.s007.pdf]

FUS WT

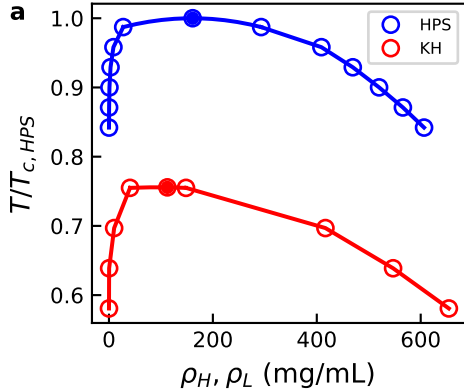

LAF-1 IDR

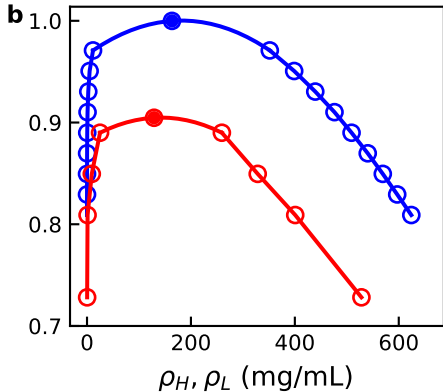

Supplement: S8 Fig — (PDF) [file pcbi.1005941.s008.pdf]

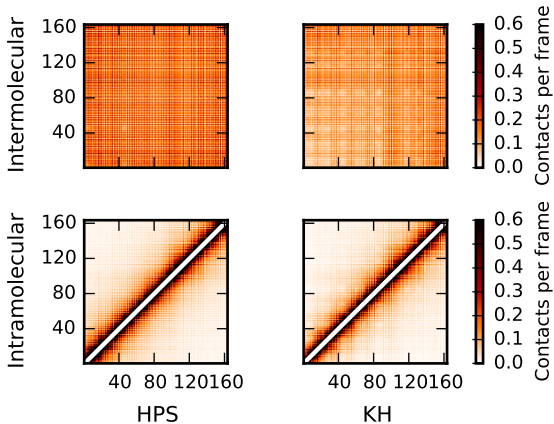

Supplement: S9 Fig — (PDF) [file pcbi.1005941.s009.pdf]

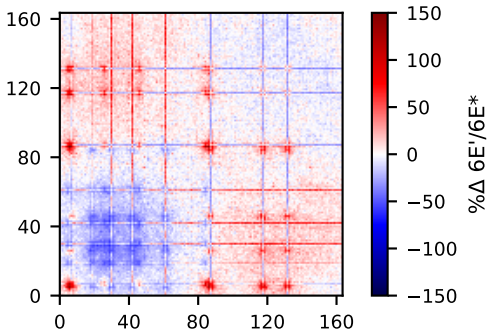

Supplement: S10 Fig — (PDF) [file pcbi.1005941.s010.pdf]

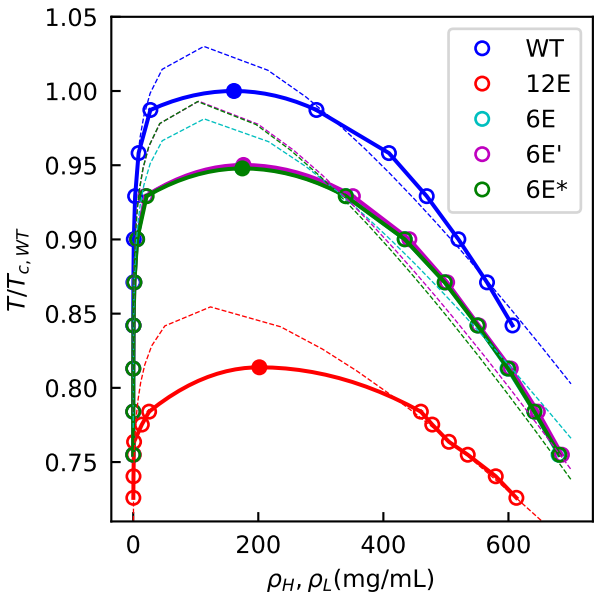

Supplement: S11 Fig — (PDF) [file pcbi.1005941.s011.pdf]

FUS

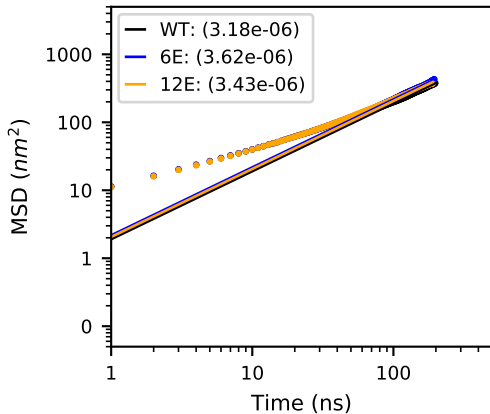

LAF-1

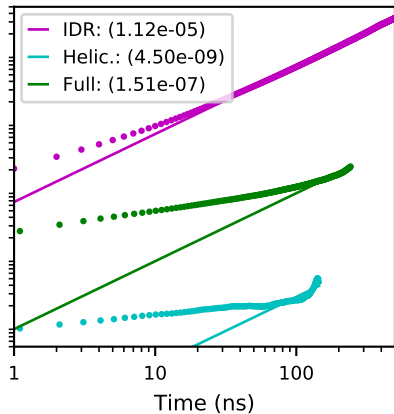

Supplement: S12 Fig — (PDF) [file pcbi.1005941.s012.pdf]

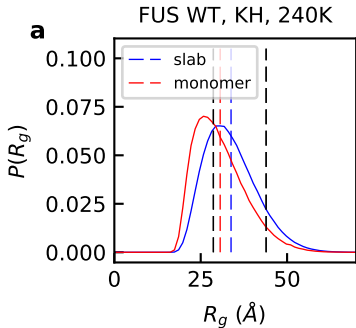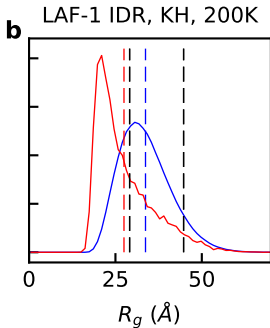

Supplement: S13 Fig — (PDF) [file pcbi.1005941.s013.pdf]

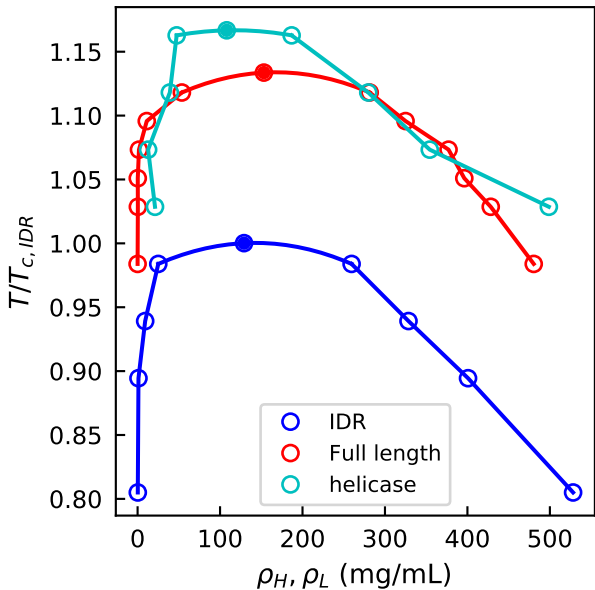

Supplement: S14 Fig — (PDF) [file pcbi.1005941.s014.pdf]

IDR

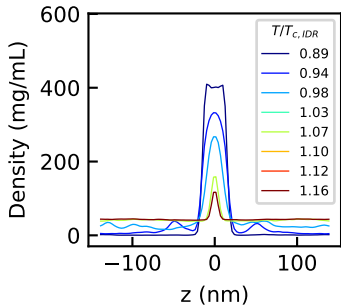

Helicase

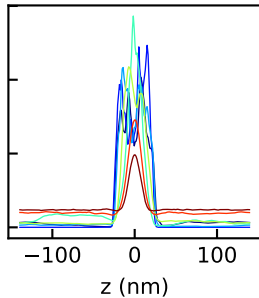

Full length

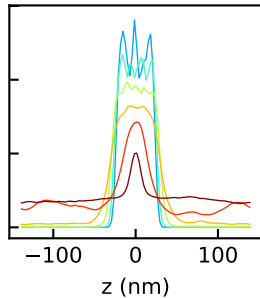

Supplement: S15 Fig — (PDF) [file pcbi.1005941.s015.pdf]

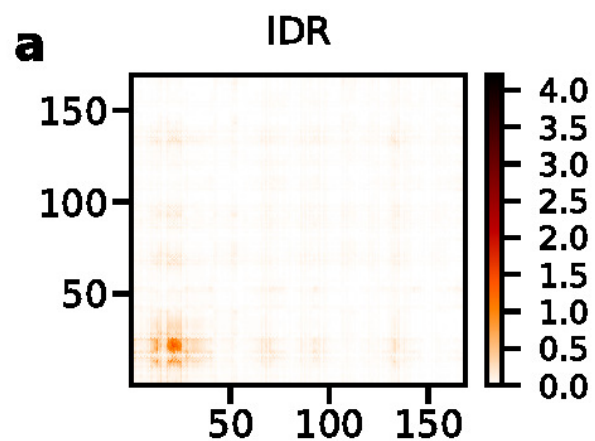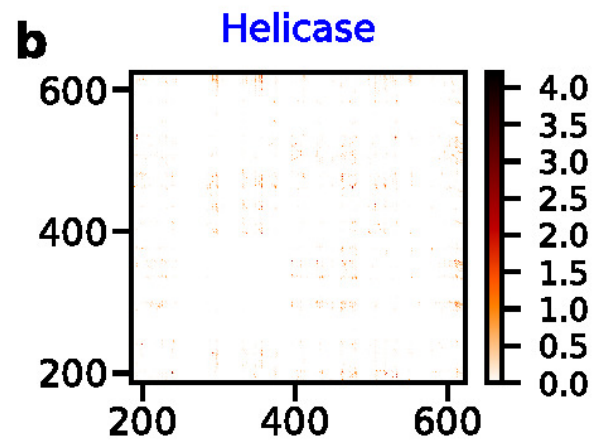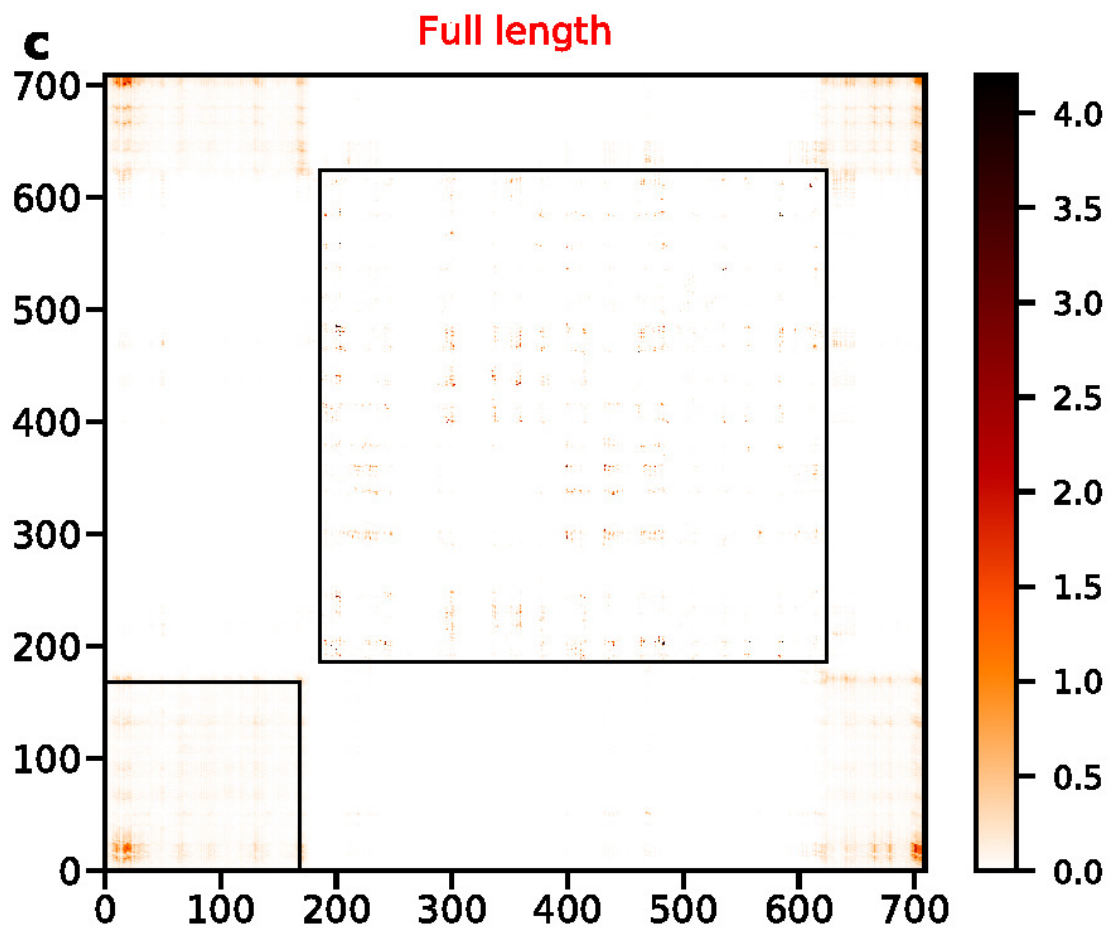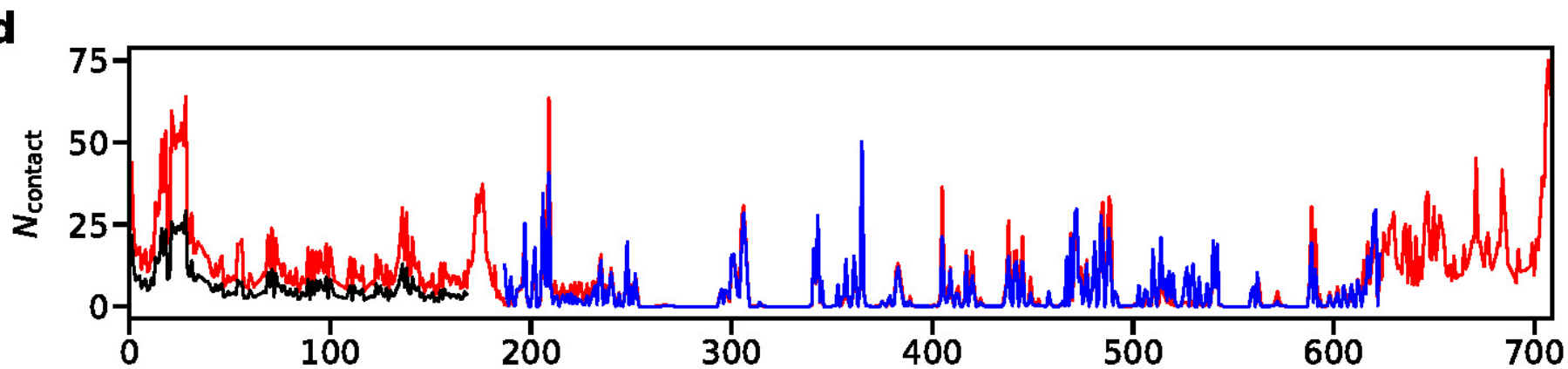

Supplement: S16 Fig — (PDF) [file pcbi.1005941.s016.pdf]

dilute phase

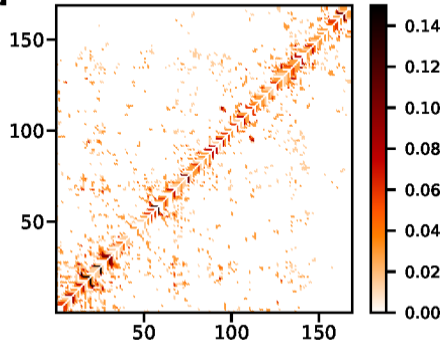

concentrated phase

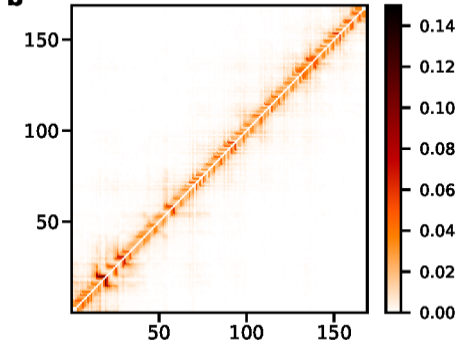

Supplement: S17 Fig — (PDF) [file pcbi.1005941.s017.pdf]

[FUS40]<sub>1</sub>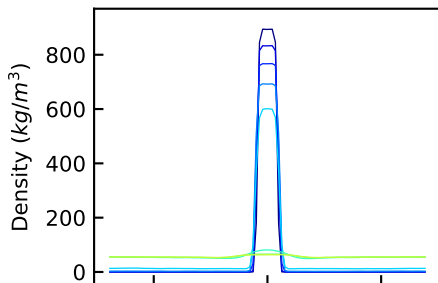[FUS40]<sub>2</sub>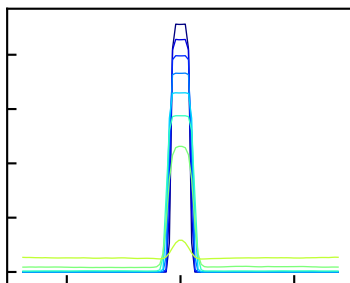[FUS40]<sub>3</sub>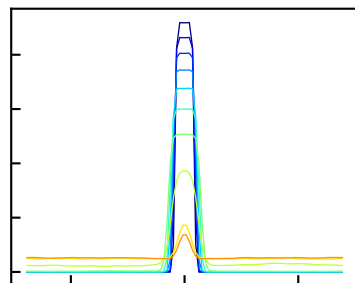[FUS40]<sub>4</sub>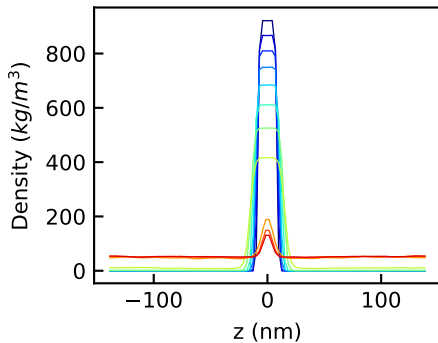[FUS40]<sub>5</sub>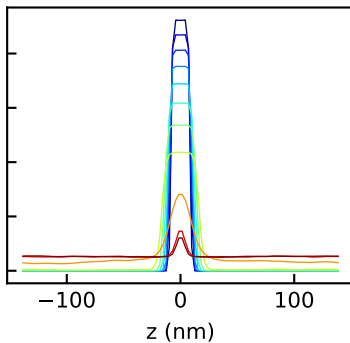 $T/T_{c, [\text{FUS40}]_1}$ 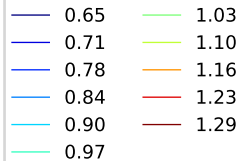

Supplement: S18 Fig — (PDF) [file pcbi.1005941.s018.pdf]

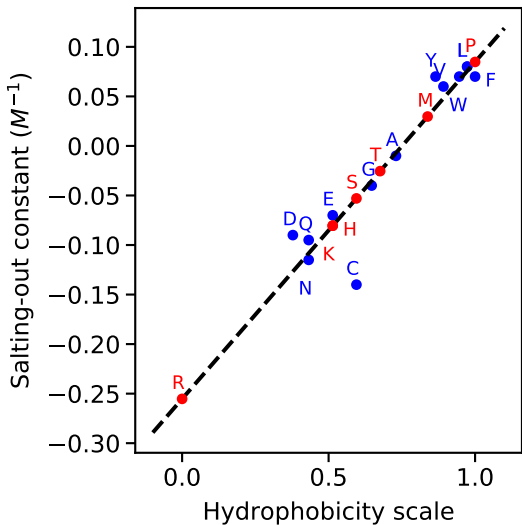

Supplement: S19 Fig — (PDF) [file pcbi.1005941.s019.pdf]

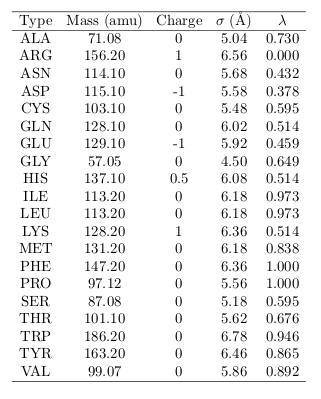

Supplement: S1 Table — (PNG) [file pcbi.1005941.s020.png]

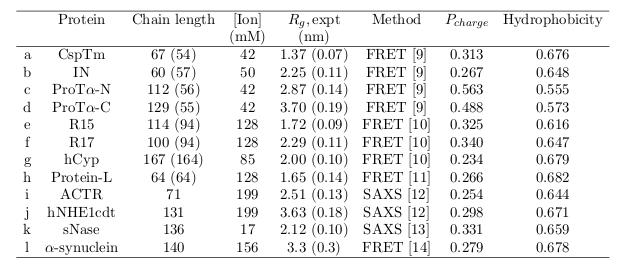

Supplement: S2 Table — (PNG) [file pcbi.1005941.s021.png]

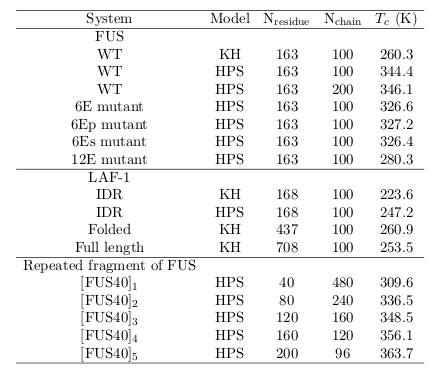

Supplement: S3 Table — (PNG) [file pcbi.1005941.s022.png]

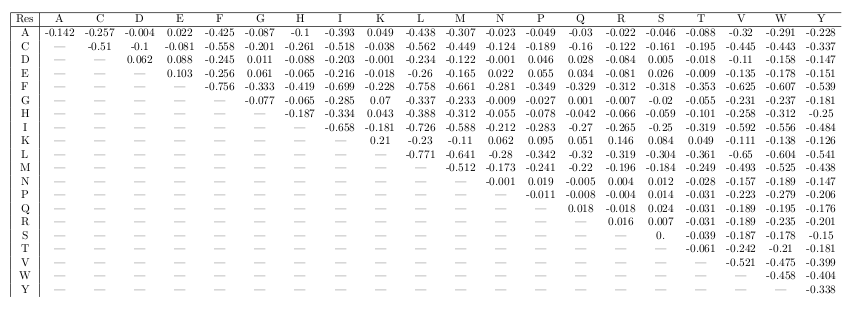

Supplement: S4 Table — (PNG) [file pcbi.1005941.s023.png]

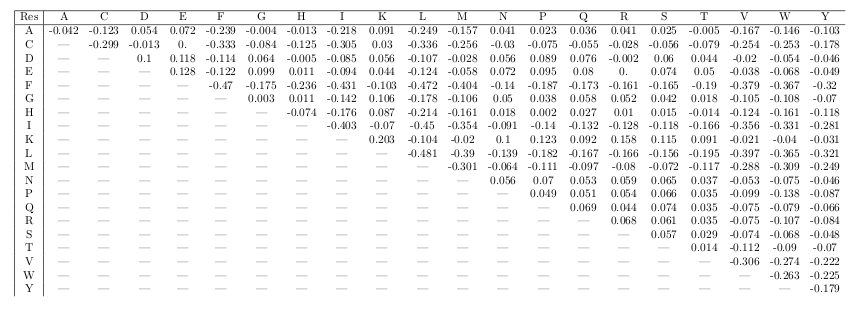

Supplement: S5 Table — (PNG) [file pcbi.1005941.s024.png]

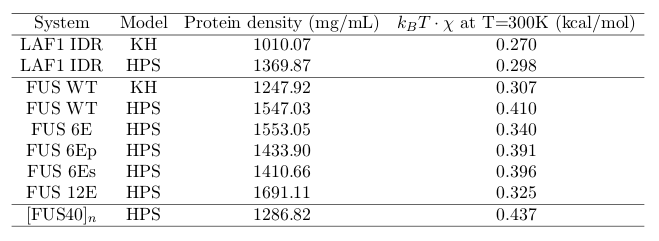

Supplement: S6 Table — (PNG) [file pcbi.1005941.s025.png]
