# Supplementary material for: Sequence determinants of protein phase behavior from a coarse-grained model
Source: PLoS Comput Biol. 2018 Jan 24;14(1):e1005941. doi: 10.1371/journal.pcbi.1005941 (PMC5798848; doi:10.1371/journal.pcbi.1005941)
Supplement: S1 Document — (PDF) [file pcbi.1005941.s036.pdf]

Supporting Information for: "Sequence determinants of protein phase behavior from a coarse-grained model"

Gregory L. Dignon[1], Wenwei Zheng[2], Young C. Kim[3], Robert B. Best[2], and Jeetain Mittal[1a]

[1]Department of Chemical and Biomolecular Engineering, Lehigh University, Bethlehem, Pennsylvania 18015, United States

[2]National Insitute of Diabetes and Digestive and Kidney Diseases, National Institutes of Health, Bethesda, 20892, United States

[3]Center for Materials Physics and Technology, Naval Research Laboratory, Washington, DC 20375, United States

[a]Electronic mail: jeetain@lehigh.edu

1. Supplementary Methods

Modelling of LAF-1 helicase domain.
Since there is no solved structure for the helicase domain of LAF-1, we started by predicting its structure from its homologue VASA. Both LAF-1 and VASA belong to the DEAD-Box family and the structure of *Drosophila* VASA has been solved [1]. We first aligned the LAF-1 sequence to the structured part of VASA using the MUSCLE v3.8 web service [2]. The sequence similarity is 51% and the alignment of the structured part is shown below:

```
VASA    Residue 202-621
LAF-1   Residue 187-623
VASA    YIPPEPSNDAIEI-FSSGIASGIHFSKYNNIPVKVTGSDVPQPIQHFTSADLRDIIIDNV
LAF-1   WENRGARDERIEQELFSGQLSGINFDKYEEIPVEATGDDVPQPISLFSDLSLHEWIEENI
        :    . :: **  : **  ***:*.**::***:.**.******. *:. .*.: * :*:
VASA    NKSGYKIPTPIQKCSIPVISSGRDLMACAQTGSGKTAAFLLPILSKLLED-PHELEL---
LAF-1   KTAGYDRPTPVQKYSIPALQGGRDLMSCAQTGSGKTAAFLVPLVNAILQDGPDAVHRSVT
        :.:**. ***:** ***.:..*****:*************:*::. :*:* *  :
VASA    ---GR----PQVVIVSPTRELAIQIFNEARKFAFESYLKIGIVYGG-TSFRHQNECITRG
LAF-1   SSGGRKKQYPSALVLSPTRELSLQIFNESRKFAYRTPITSALLYGGRENYKDQIHKLRLG
           **    *..:::******::*****:****: : :. .::*** .:.  *   :  *
VASA    CHVVIATPGRLLDFVDRTFITFEDTRFVVLDEADRMLDMGFSEDMRRIM--THVTMRPEH
LAF-1   CHILIATPGRLIDVMDQGLIGMEGCRYLVLDEADRMLDMGFEPQIRQIVECNRMPSKEER
        **::*******:*.:*. :* :*. *::*************. ::*.*:  ..:. . *.
VASA    QTLMFSATFPEEIQRMAGEFLK-NYVFVAIGIVGGACSDVKQTIYEVNKYAKRSKLIEIL
LAF-1   ITAMFSATFPKEIQLLAQDFLKENYVFLAVGRVGSTSENIMQKIVWVEEDEKRSYLMDLL
         * *******:*** :* :*** ****:*:* **.:..:: *.*  *::  *** *:::*
VASA    SEQADG--TIVFVETKRGADFLASFLSEKEFPTTSIHGDRLQSQREQALRDFKNGSMKVL
LAF-1   DATGDSSLTLVFVETKRGASDLAYYLNRQNYEVVTIHGDLKQFEREKHLDLFRTGTAPIL
        .  .*.  *:*********. ** :*. ::: ..:****  * :**: *  *..*:  :*
VASA    IATSVASRGLDIKNIKHVINYDMPSKIDDYVHRIGRTGRVGNNGRATSFFDPEKDRAIAA
LAF-1   VATAVAARGLDIPNVKHVINYDLPSDVDEYVHRIGRTGRVGNVGLATSFFN-DKNRNIAR
        :**:**:***** *:*******:**.:*:************* * *****: :*:* **
VASA    DLVKILEGSGQTVPDFLR
LAF-1   ELMDLIVEANQELPDWLE
        :*:.::  :.* :**:*
```

We provided the VASA structure (PDB:2DB3) and the sequence alignment information shown above as the inputs to the Modeller software package v9.17 [3]. We then repeated the modelling process 100 times and picked the structure with the smallest energy as best model for the structure of the LAF-1 helicase domain (S7 Fig.).

Sequences of the proteins used in this work.
FUS WT

```
MASNDYTQQA TQSYGAYPTQ PGQGYSQQSS QPYGQQSYSG YSQSTDTSGY GQSSYSSYGQ
SQNTGYGTQS TPQGYGSTGG YGSSQSSQSS YGQQSSYPGY GQQPAPSSTS GSYGSSSQSS
```

SYGQPQSGSY SQQPSYGGQQ QSYGQQQSYN PPQGYGQQNQ YNS

FUS 6E

MASNDYTQQA TQSYGAYPTQ PGQGYEQQSE QPYGQQSYSG YSQSTDTSGY GQSSYSSYGQ
SQNTGYGEQS TPQGYGSTGG YGSEQSEQSS YGQQSSYPGY GQQPAPSSTS GSYGSSEQSS
SYGQPQSGSY SQQPSYGGQQ QSYGQQQSYN PPQGYGQQNQ YNS

FUS 6E'

MASNDYTQQA TQSYGAYPEQ PGQGYEQQSE QPYGQQSYSG YEQSTDTSGY GQSSYSSYGQ
EQNTGYGTQS TPQGYGSTGG YGSEQSSQSS YGQQSSYPGY GQQPAPSSTS GSYGSSSQSS
SYGQPQSGSY SQQPSYGGQQ QSYGQQQSYN PPQGYGQQNQ YNS

FUS 6E*

MASNDYEQQA TQSYGAYPTQ PGQGYEQQSS QPYGQQSYSG YSQSTDTSGY GQSSYSSYGQ
SQNTGYGTQS TPQGYGSTGG YGSEQSEQSS YGQQSSYPGY GQQPAPSSTS GSYGSSEQSS
SYGQPQSGSY EQQPSYGGQQ QSYGQQQSYN PPQGYGQQNQ YNS

FUS 12E

MASNDYEQQA EQSYGAYPEQ PGQGYEQQSE QPYGQQSYSG YEQSTDTSGY GQSSYSSYGQ
EQNTGYGEQS TPQGYGSTGG YGSEQSEQSS YGQQSSYPGY GQQPAPSSTS GSYGSSEQSS
SYGQPQSGSY EQQPSYGGQQ QSYGQQQSYN PPQGYGQQNQ YNS

LAF-1 IDR

MESNQSNNGG SGNAALNRGG RYVPPHLRGG DGGAAAAASA GGDDRRGGAG GGGYRRGGGN
SGGGGGGGYD RGYNDNRDDR DNRGGSGGYG RDRNYEDRGY NGGGGGGGNR GYNNNRGGGG
GGYNRQDRGD GGSSNFSRGG YNNRDEGSDN RGSGRSYNND RRDNGGDG

LAF-1 full length

...QN TRWNNLDAPP
SRGTSKWENR GARDERIEQE LFSGQLSGIN FDKYEEIPVE ATGDDVPQPI SLFSDLSLHE
WIEENIKTAG YDRPTPVQKY SIPALQGGRD LMSCAQTGSG KTAAFLVPLV NAILQDGPDA
VHRSVTSSGG RKKQYPSALV LSPTRELSLQ IFNESRKFAY RTPITSALLY GGRENYKDQI
HKLRLGCHIL IATPGRLIDV MDQGLIGMEG CRYLVLDEAD RMLDMGFEPQ IRQIVECNRM
PSKEERITAM FSATFPKEIQ LLAQDFLKEN YVFLAVGRVG STSENIMQKI VWVEEDEKRS
YLMDLLDATG DSSLTLVFVE TKRGASDLAY YLNRQNYEVV TIHGDLKQFE REKHLDLFRT
GTAPILVATA VAARGLDIPN VKHVINYDLP SDVDEYVHRI GRTGRVGNVG LATSFFNDKN
RNIARELMDL IVEANQELPD WLE

CspTm

GPGMRGKVKW FDSKKGYGFI TKDEGGDVFV HWSAIEMEGF KTLKEGQVVE FEIQEGKKGG
QAAHVKV

IN

GSHCFLDGID KAQEEHEKYH SNWRAMASDF NLPPVVAKEI VASCDKCQLK GEAMHGQVDC

ProTα-N

GPSDAAVDTS SEITTKDLKE KKEVVEEAEN GRDAPANGNA ENEENGEQEA DNEVDEECEE
GGEEEEEEEE GDGEEEDGDE DEEAESATGK RAAEDDEDDD VDTKKQKTDE DD

ProTα-C

MAHHHHHHSA ALEVLFQGPM SDAAVDTSSE ITTKDLKEKK EVVEEAENGR DAPANGNANE ENGEQEADNE VDEECEEGGE EEEEEEEGDG EEEDGDEDEE AESATGKRAA EDDEDDDVDT KKQKTDEDD

R15

KLKEANKQQN FNTGIKDFDF WLSEVEALLA SEDYGKDLAS VNNLLKKHQL LEADISAHED RLKDLNSQAD SLMTSSAFDT SQVKDKRETI NGRFQRIKSM AAARRAKLNE SHRL

R17

RLEESLEYQQ FVANVEEEEA WINEKMTLVA SEDYGDTLAA IQGLLKKHEA FETDFTVHKD RVNDVAANGE DLIKKNNHHV ENITAKMKGL KGKVSDLEKA

hCyp

SSFHRIIPGF MSQGGDFTRH NGTGGKSIYG EKFEDENFIL KHTGPGILSM ANAGPNTNGS QFFISTAKTE FLDGKHVVFG KVKEGMNIVE AMERFGSRNG KTSKKITIAD SGQLE

Protein L

MEEVTIKANL IFANGSTQTA EFKGTFEKAT SEAYAYADTL KKDNGEWTVD VADKGYTLNI KFAG

ACTR

GTQNRPLLRN SLDDLVGPPS NLEGQSDERA LLDQLHTLLS NTDATGLEEI DRALGIPELV NQGQALEPKQ D

hNHE1cdt

MVPAHKLDSP TMSRARIGSD PLAYEPKEDL PVITIDPASP QSPESVDLVN EELKGKVLGL SRDPAKVAEE DEDDDGGIMM RSKETSSPGT DDVFTPAPSD SPSSQRIQRC LSDPGPHPEP GEGEPFFPKG Q

sNase

ATSTKKLHKE PATLIKAIDG DTVKLMYKGQ PMTFRLLLVD TPETKHPKKG VEKYGPEASA FTKKMVENAK KIEVEFDKGQ RTDKYGRGLA YIYADGKMVN EALVRQGLAK VAYVYKPNNT HEQHLRKSEA QAKKEK

α-synuclein

MDVFMKGLSK AKEGVVAAAE KTKQGVAEAA GKTKEGVLYV GSKTKEGVVH GVATVAEKTK EQVTNVGGAV VTGVTAVAQK TVEGAGSIAA ATGFVKKDQL GKNEEGAPQE GILEDMPVDP DNEAYEMPSE EGYQDYEPEA

2. Supplementary Figures

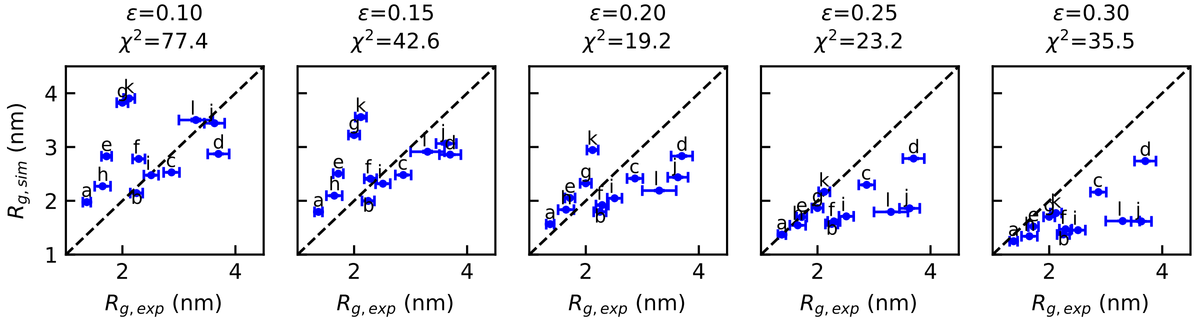

Figure S1. Comparison of R_g between simulations and experiments with different ϵ parameters for HPS model. The deviations χ^2 between the simulations and experiments are shown in the title. The list of the proteins and legends can be found in S2 Table.

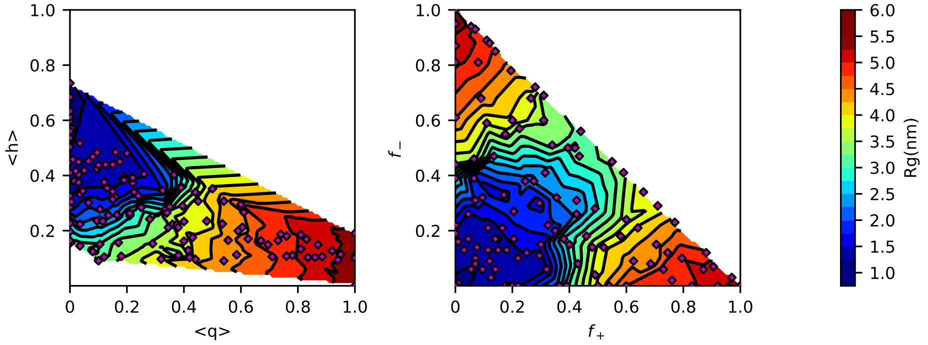

Figure S2. Randomly generated 100-mers in a Uversky (left) and Pappu (right) plot to show the dependence of R_g on charge and hydrophobicity using the KH model.

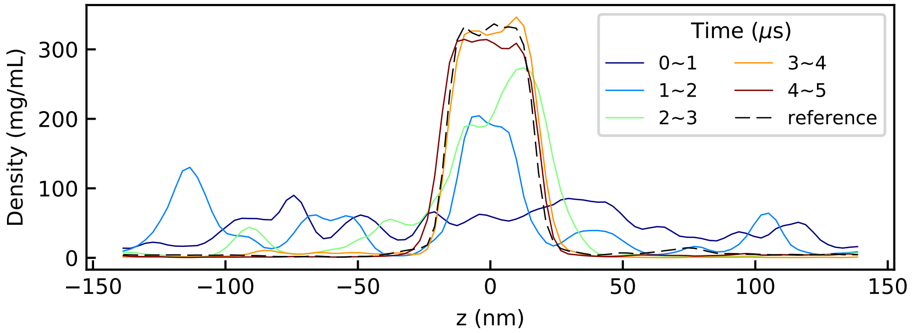

Figure S3. LAF-1 simulation started from dispersed state at 210K with KH-D model showing coalescence to a slab conformation after about 4 μs. The colored lines show the density profile at different time ranges throughout the simulation. The black line shows the simulation starting from an initial slab configuration as a reference.

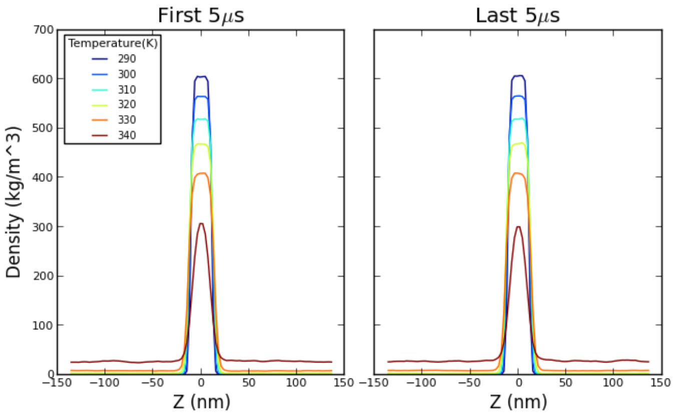

Figure S4. Comparison of density profiles between first 5μs and last 5μs of slab simulations of FUS WT.

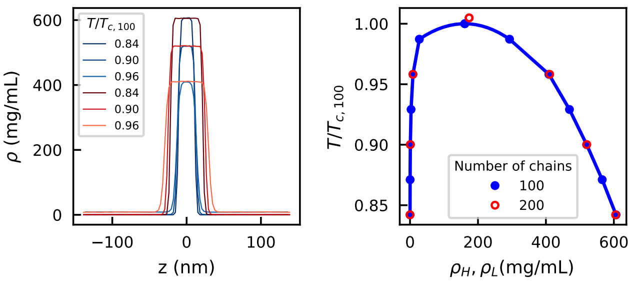

Figure S5. Comparison of FUS WT simulations with 100 (blue) and 200 (red) chains. Temperatures are scaled by the critical temperature of the simulations with 100 chains.

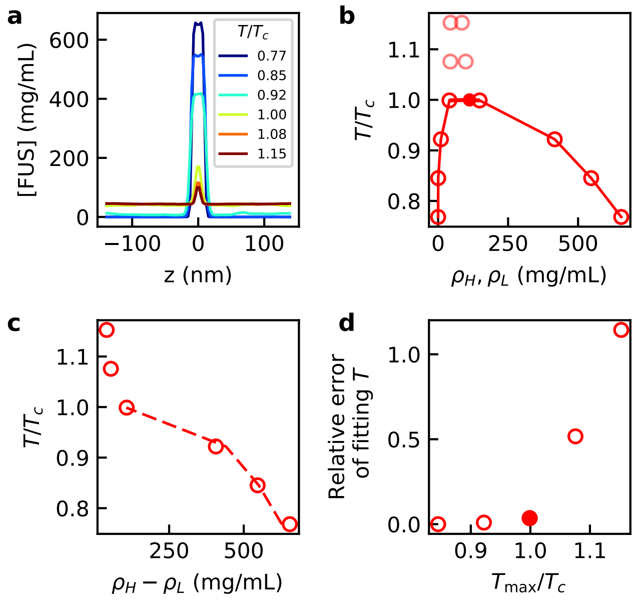

Figure S6. Methodology used to determine the range of temperatures to fit Eq. 6 in the main text. Temperatures are scaled by the critical temperature. a) Dependence of WT FUS concentration on the z-axis of the SLAB simulation with KH model. b) The concentrations of low- and high-density phases as a function of temperature. The empty symbols show the data and the solid symbol shows the critical temperature obtained from fitting to all except the two highest temperatures, where the system cannot be described by Eq. 6 any more. c) The difference of concentrations between the low- and high-density phases as a function of the temperature. Dashed line shows the fitting to Eq. 6. d) The relative error of fitting T using Eq. 6 as a function of the maximum temperature for fitting. The maximum temperature used for fitting to obtain the critical temperature is shown in solid symbol.

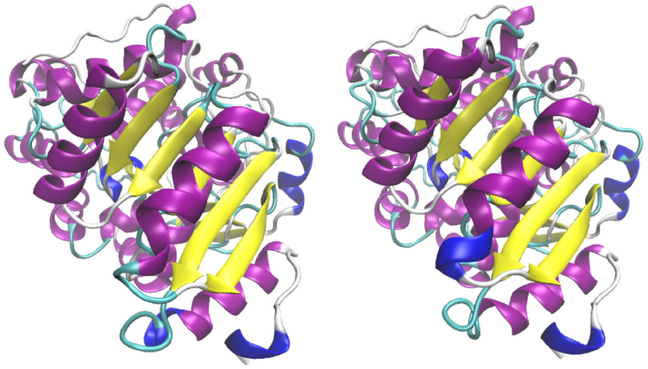

Figure S7. Homology modelling of helicase domain of LAF-1 using the structure of VASA. Left: the structure of VASA residue 202-621 (PDB:2DB3[1]); Right: the structure of LAF-1 helicase domain residue 187-623 from homology modelling.

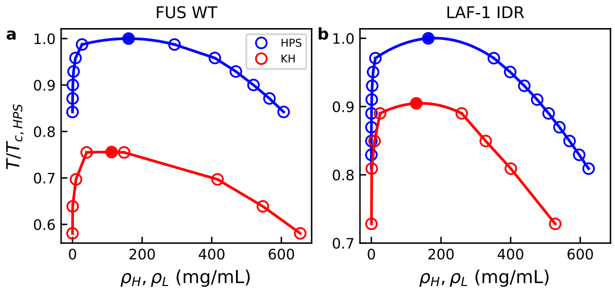

Figure S8. Comparison of the phase diagram generated with HPS (blue) and KH (red) model in FUS WT (left) and LAF-1 IDR (right). Temperatures are scaled by critical temperatures using HPS model. Dashed lines show the fit to the Flory-Huggins theory.

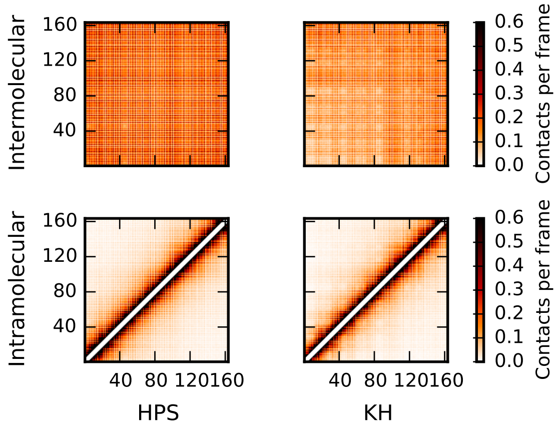

Figure S9. Inter- (upper) and intra-molecular (lower) contact maps for FUS WT at 260 K using HPS (left) and KH models (right).

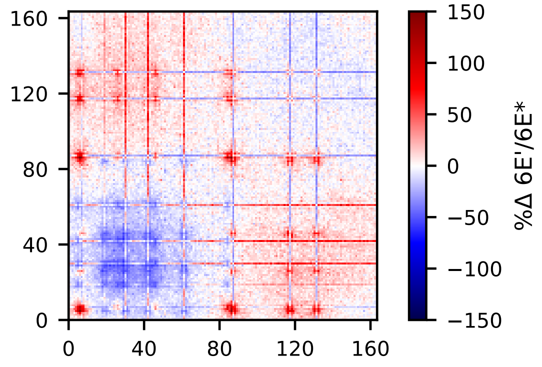

Figure S10. Intermolecular contacts for FUS 6E' divided by that of FUS 6E* showing how the overall number of contacts forming within the slab changes between the two sequences.

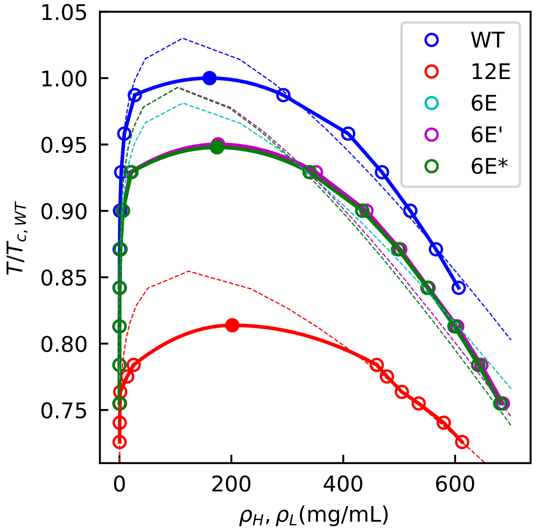

Figure S11. Phase diagram for FUS WT, 6E variants and 12E fitting to the Flory-Huggins theory. Temperatures are scaled by the critical temperature of FUS WT. Dashed lines show the fit to the Flory-Huggins theory.

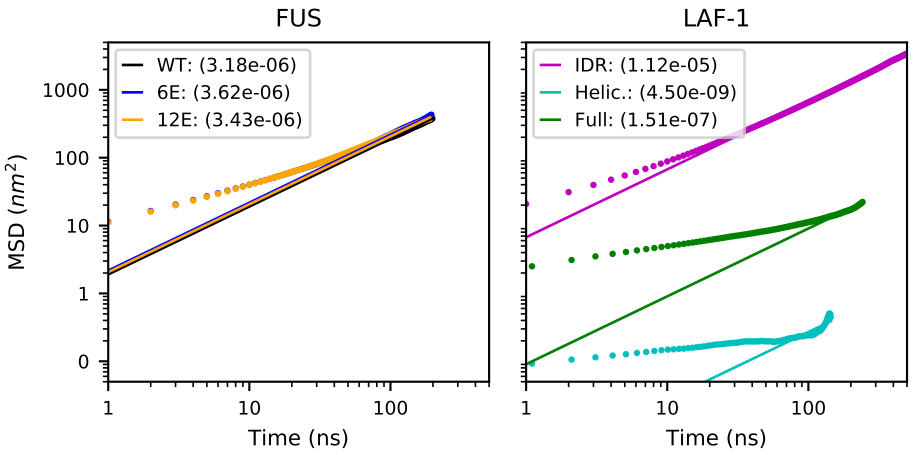

Figure S12. Mean squared displacement (MSD) as a function of time for FUS variants at 260K and 600 mg/mL (left), and LAF-1 at 210K and 260, 535 and 500 mg/mL for IDR, helicase, and full length respectively. Linear fits were calculated using all data points after 100 ns. Diffusion coefficients are included in parentheses in units of cm^2/s .

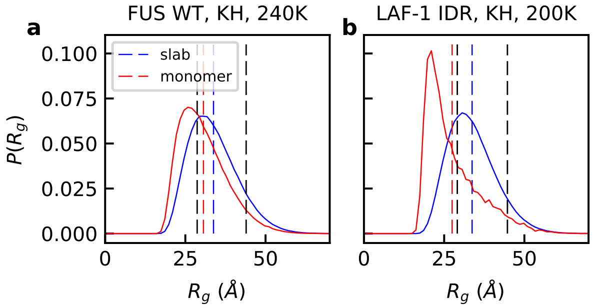

FIGURE S13. Radii of gyration of the disordered proteins inside (blue) and out of (red) the slab. a) FUS WT with KH model at 240K. b) LAF-1 IDR with KH model at 200K. Black lines show the R_g from the random coil or excluded volume chain with the same chain length.

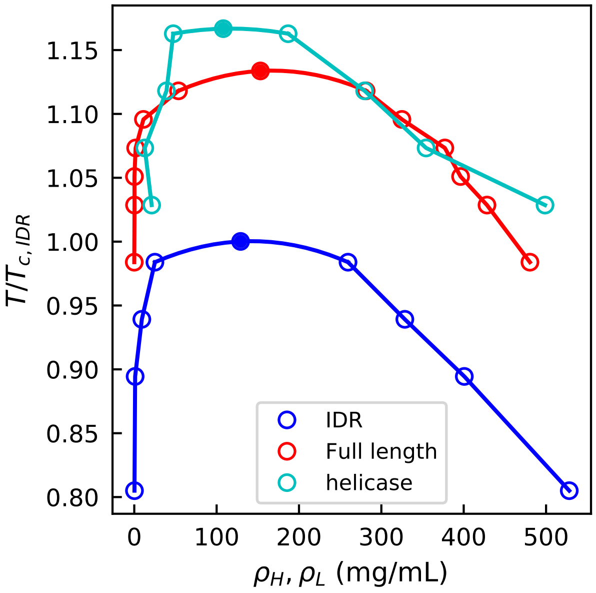

FIGURE S14. Phase diagram of IDR (blue), helicase (cyan) and full length (red) LAF-1. Temperatures are scaled by the critical temperature of IDR LAF-1.

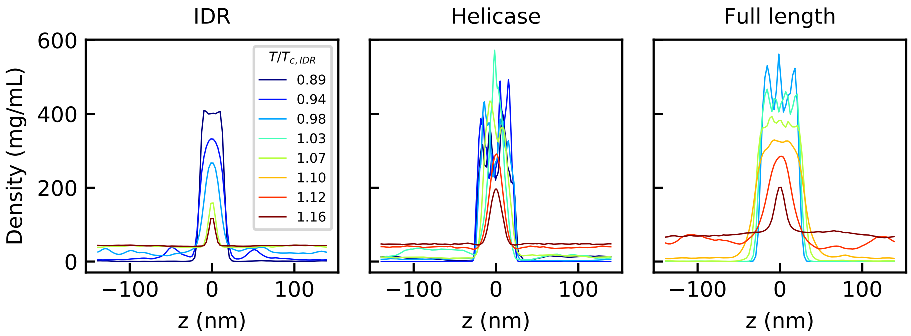

Figure S15. Slab density profiles of IDR (left), helicase (middle) or full length (right) LAF-1. Temperatures are scaled by the critical temperature of IDR LAF-1.

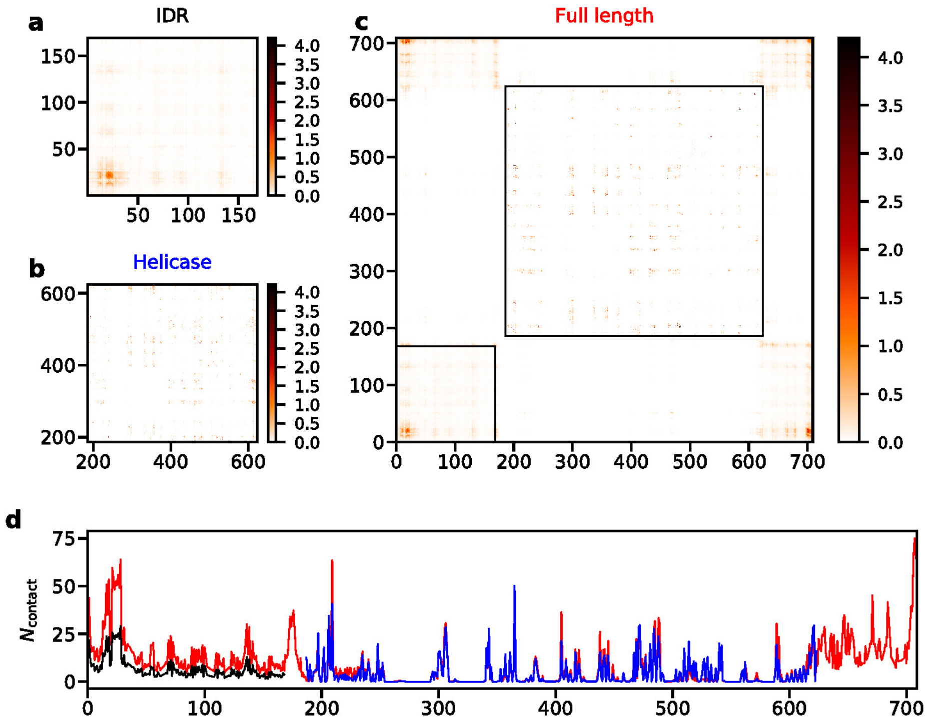

FIGURE S16. Number of intermolecular contacts per frame for different LAF-1 variants at 220K. a) Contact map of IDR LAF-1. b) Contact map of helicase LAF-1. c) Contact map of full length LAF-1. Black boxes illustrate the N-terminal IDR and the helicase domain. d) Number of intermolecular contacts per residue per frame for IDR LAF-1 (black), helicase LAF-1 (blue) and full length LAF-1 (red).

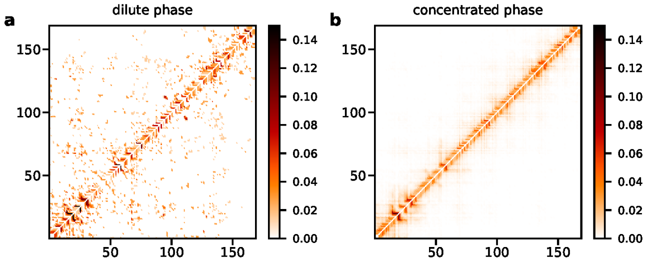

Figure S17. Number of intramolecular contacts per frame for LAF1 IDR with KH model at 200K. The contact map for the chains in the dilute phase is shown on the left side and that in the concentrated phase is shown on the right side.

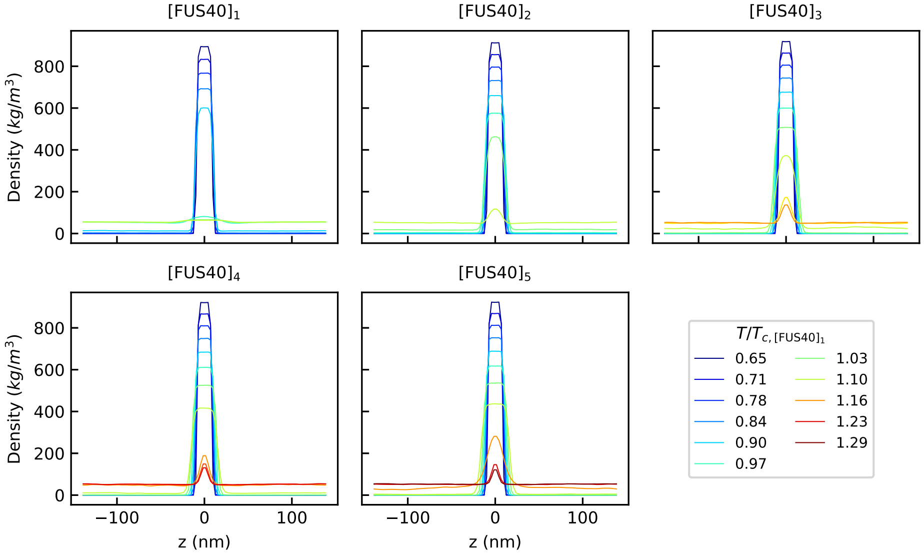

Figure S18. Slab density profiles of the repeated peptides of FUS fragment. Temperatures are scaled by the critical temperature of $[\mathrm{FUS40}]_1$.

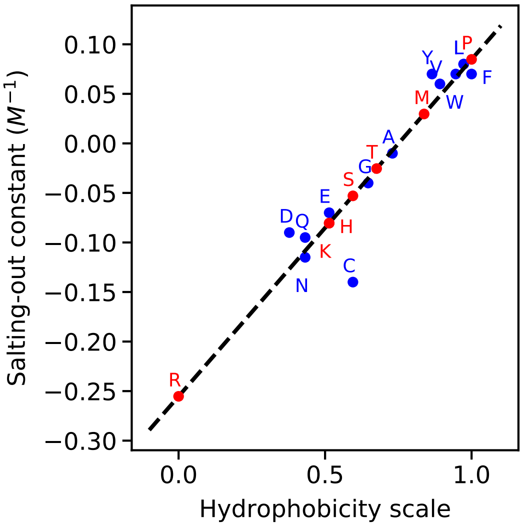

FIGURE S19. The correlation between salting-out constant and hydrophobicity scale. Black line shows the linear fitting curve between these two parameters. Blue dots show the data from literature [4, 5, 6, 7] and red dots show the estimate from linear inter- or extrapolation.

3. Supplementary Tables

Table S1. The amino acid parameters used in the HPS model. σ is the diameter of the amino acid used in the short-ranged pair potential. λ is the scaled hydrophobicity from the literature [8].

Type	Mass (amu)	Charge	σ (Å)	λ
ALA	71.08	0	5.04	0.730
ARG	156.20	1	6.56	0.000
ASN	114.10	0	5.68	0.432
ASP	115.10	-1	5.58	0.378
CYS	103.10	0	5.48	0.595
GLN	128.10	0	6.02	0.514
GLU	129.10	-1	5.92	0.459
GLY	57.05	0	4.50	0.649
HIS	137.10	0.5	6.08	0.514
ILE	113.20	0	6.18	0.973
LEU	113.20	0	6.18	0.973
LYS	128.20	1	6.36	0.514
MET	131.20	0	6.18	0.838
PHE	147.20	0	6.36	1.000
PRO	97.12	0	5.56	1.000
SER	87.08	0	5.18	0.595
THR	101.10	0	5.62	0.676
TRP	186.20	0	6.78	0.946
TYR	163.20	0	6.46	0.865
VAL	99.07	0	5.86	0.892

Table S2. List of intrinsically disordered or unfolded proteins with experimentally determined R_g. The radii of gyration of ACTR and hNHE1cdt were measured at 5°C and 45°C in the experiment and have been interpolated to 25°C for comparison with the other proteins.

	Protein	Chain length	[Ion] (mM)	R_g, expt (nm)	Method	P_{charge}	Hydrophobicity
a	CspTm	67 (54)	42	1.37 (0.07)	FRET [9]	0.313	0.676
b	IN	60 (57)	50	2.25 (0.11)	FRET [9]	0.267	0.648
c	ProTα-N	112 (56)	42	2.87 (0.14)	FRET [9]	0.563	0.555
d	ProTα-C	129 (55)	42	3.70 (0.19)	FRET [9]	0.488	0.573
e	R15	114 (94)	128	1.72 (0.09)	FRET [10]	0.325	0.616
f	R17	100 (94)	128	2.29 (0.11)	FRET [10]	0.340	0.647
g	hCyp	167 (164)	85	2.00 (0.10)	FRET [10]	0.234	0.679
h	Protein-L	64 (64)	128	1.65 (0.14)	FRET [11]	0.266	0.682
i	ACTR	71	199	2.51 (0.13)	SAXS [12]	0.254	0.644
j	hNHE1cdt	131	199	3.63 (0.18)	SAXS [12]	0.298	0.671
k	sNase	136	17	2.12 (0.10)	SAXS [13]	0.331	0.659
l	α-synuclein	140	156	3.3 (0.3)	FRET [14]	0.279	0.678

Table S3. Interaction parameters (ϵ_{ij}) used for KH-D model [15] (kcal/mol)

Res	A	C	D	E	F	G	H	I	K	L	M	N	P	Q	R	S	T	V	W	Y
A	-0.142	-0.257	-0.004	0.022	-0.425	-0.087	-0.1	-0.393	0.049	-0.438	-0.307	-0.023	-0.049	-0.03	-0.022	-0.046	-0.088	-0.32	-0.291	-0.228
C	—	-0.51	-0.1	-0.081	-0.558	-0.201	-0.261	-0.518	-0.038	-0.562	-0.449	-0.124	-0.189	-0.16	-0.122	-0.161	-0.195	-0.445	-0.443	-0.337
D	—	—	0.062	0.088	-0.245	0.011	-0.088	-0.203	-0.001	-0.234	-0.122	-0.001	0.046	0.028	-0.084	0.005	-0.018	-0.11	-0.158	-0.147
E	—	—	—	0.103	-0.256	0.061	-0.065	-0.216	-0.018	-0.26	-0.165	0.022	0.055	0.034	-0.081	0.026	-0.009	-0.135	-0.178	-0.151
F	—	—	—	—	-0.756	-0.333	-0.419	-0.699	-0.228	-0.758	-0.661	-0.281	-0.349	-0.329	-0.312	-0.318	-0.353	-0.625	-0.607	-0.539
G	—	—	—	—	—	-0.077	-0.065	-0.285	0.07	-0.337	-0.233	-0.009	-0.027	0.001	-0.007	-0.02	-0.055	-0.231	-0.237	-0.181
H	—	—	—	—	—	—	-0.187	-0.334	0.043	-0.388	-0.312	-0.055	-0.078	-0.042	-0.066	-0.059	-0.101	-0.258	-0.312	-0.25
I	—	—	—	—	—	—	—	-0.658	-0.181	-0.726	-0.588	-0.212	-0.283	-0.27	-0.265	-0.25	-0.319	-0.592	-0.556	-0.484
K	—	—	—	—	—	—	—	—	0.21	-0.23	-0.11	0.062	0.095	0.051	0.146	0.084	0.049	-0.111	-0.138	-0.126
L	—	—	—	—	—	—	—	—	—	-0.771	-0.641	-0.28	-0.342	-0.32	-0.319	-0.304	-0.361	-0.65	-0.604	-0.541
M	—	—	—	—	—	—	—	—	—	—	-0.512	-0.173	-0.241	-0.22	-0.196	-0.184	-0.249	-0.493	-0.525	-0.438
N	—	—	—	—	—	—	—	—	—	—	—	-0.001	0.019	-0.005	0.004	0.012	-0.028	-0.157	-0.189	-0.147
P	—	—	—	—	—	—	—	—	—	—	—	—	-0.011	-0.008	-0.004	0.014	-0.031	-0.223	-0.279	-0.206
Q	—	—	—	—	—	—	—	—	—	—	—	—	—	0.018	-0.018	0.024	-0.031	-0.189	-0.195	-0.176
R	—	—	—	—	—	—	—	—	—	—	—	—	—	—	0.016	0.007	-0.031	-0.189	-0.235	-0.201
S	—	—	—	—	—	—	—	—	—	—	—	—	—	—	—	0.	-0.039	-0.187	-0.178	-0.15
T	—	—	—	—	—	—	—	—	—	—	—	—	—	—	—	—	-0.061	-0.242	-0.21	-0.181
V	—	—	—	—	—	—	—	—	—	—	—	—	—	—	—	—	—	-0.521	-0.475	-0.399
W	—	—	—	—	—	—	—	—	—	—	—	—	—	—	—	—	—	—	-0.458	-0.404
Y	—	—	—	—	—	—	—	—	—	—	—	—	—	—	—	—	—	—	—	-0.338

Table S4. Interaction parameters (ϵ_{ij}) used for KH-A model [15] (kcal/mol)

Res	A	C	D	E	F	G	H	I	K	L	M	N	P	Q	R	S	T	V	W	Y
A	-0.042	-0.123	0.054	0.072	-0.239	-0.004	-0.013	-0.218	0.091	-0.249	-0.157	0.041	0.023	0.036	0.041	0.025	-0.005	-0.167	-0.146	-0.103
C	—	-0.299	-0.013	0.	-0.333	-0.084	-0.125	-0.305	0.03	-0.336	-0.256	-0.03	-0.075	-0.055	-0.028	-0.056	-0.079	-0.254	-0.253	-0.178
D	—	—	0.1	0.118	-0.114	0.064	-0.005	-0.085	0.056	-0.107	-0.028	0.056	0.089	0.076	-0.002	0.06	0.044	-0.02	-0.054	-0.046
E	—	—	—	0.128	-0.122	0.099	0.011	-0.094	0.044	-0.124	-0.058	0.072	0.095	0.08	0.	0.074	0.05	-0.038	-0.068	-0.049
F	—	—	—	—	-0.47	-0.175	-0.236	-0.431	-0.103	-0.472	-0.404	-0.14	-0.187	-0.173	-0.161	-0.165	-0.19	-0.379	-0.367	-0.32
G	—	—	—	—	—	0.003	0.011	-0.142	0.106	-0.178	-0.106	0.05	0.038	0.058	0.052	0.042	0.018	-0.105	-0.108	-0.07
H	—	—	—	—	—	—	-0.074	-0.176	0.087	-0.214	-0.161	0.018	0.002	0.027	0.01	0.015	-0.014	-0.124	-0.161	-0.118
I	—	—	—	—	—	—	—	-0.403	-0.07	-0.45	-0.354	-0.091	-0.14	-0.132	-0.128	-0.118	-0.166	-0.356	-0.331	-0.281
K	—	—	—	—	—	—	—	—	0.203	-0.104	-0.02	0.1	0.123	0.092	0.158	0.115	0.091	-0.021	-0.04	-0.031
L	—	—	—	—	—	—	—	—	—	-0.481	-0.39	-0.139	-0.182	-0.167	-0.166	-0.156	-0.195	-0.397	-0.365	-0.321
M	—	—	—	—	—	—	—	—	—	—	-0.301	-0.064	-0.111	-0.097	-0.08	-0.072	-0.117	-0.288	-0.309	-0.249
N	—	—	—	—	—	—	—	—	—	—	—	0.056	0.07	0.053	0.059	0.065	0.037	-0.053	-0.075	-0.046
P	—	—	—	—	—	—	—	—	—	—	—	—	0.049	0.051	0.054	0.066	0.035	-0.099	-0.138	-0.087
Q	—	—	—	—	—	—	—	—	—	—	—	—	—	0.069	0.044	0.074	0.035	-0.075	-0.079	-0.066
R	—	—	—	—	—	—	—	—	—	—	—	—	—	—	0.068	0.061	0.035	-0.075	-0.107	-0.084
S	—	—	—	—	—	—	—	—	—	—	—	—	—	—	—	0.057	0.029	-0.074	-0.068	-0.048
T	—	—	—	—	—	—	—	—	—	—	—	—	—	—	—	—	0.014	-0.112	-0.09	-0.07
V	—	—	—	—	—	—	—	—	—	—	—	—	—	—	—	—	—	-0.306	-0.274	-0.222
W	—	—	—	—	—	—	—	—	—	—	—	—	—	—	—	—	—	—	-0.263	-0.225
Y	—	—	—	—	—	—	—	—	—	—	—	—	—	—	—	—	—	—	—	-0.179

Table S5. Summary of slab simulations and critical temperatures obtained.

System	Model	$N_{residue}$	N_{chain}	T_c (K)
FUS				
WT	KH	163	100	260.3
WT	HPS	163	100	344.4
WT	HPS	163	200	346.1
6E mutant	HPS	163	100	326.6
6Ep mutant	HPS	163	100	327.2
6Es mutant	HPS	163	100	326.4
12E mutant	HPS	163	100	280.3
LAF-1				
IDR	KH	168	100	223.6
IDR	HPS	168	100	247.2
Folded	KH	437	100	260.9
Full length	KH	708	100	253.5
Repeated fragment of FUS				
$[FUS40]_1$	HPS	40	480	309.6
$[FUS40]_2$	HPS	80	240	336.5
$[FUS40]_3$	HPS	120	160	348.5
$[FUS40]_4$	HPS	160	120	356.1
$[FUS40]_5$	HPS	200	96	363.7

TABLE S6. List of parameters for fitting to Flory-Huggins theory.

System	Model	Protein density (mg/mL)	$k_B T \cdot \chi$ at T=300K (kcal/mol)
LAF1 IDR	KH	1010.07	0.270
LAF1 IDR	HPS	1369.87	0.298
FUS WT	KH	1247.92	0.307
FUS WT	HPS	1547.03	0.410
FUS 6E	HPS	1553.05	0.340
FUS 6Ep	HPS	1433.90	0.391
FUS 6Es	HPS	1410.66	0.396
FUS 12E	HPS	1691.11	0.325
$[\mathrm{FUS40}]_n$	HPS	1286.82	0.437

References

[1] Sengoku T, Nureki O, Nakamura A, Kobayashi S, Yokoyama S. Structural basis for RNA unwinding by the DEAD-box protein Drosophila Vasa. Cell. 2006;125(2):287–300.

[2] Edgar RC. MUSCLE: multiple sequence alignment with high accuracy and high throughput. Nucleic Acids Res. 2004;32(5):1792–1797.

[3] Šali A, Blundell TL. Comparative protein modelling by satisfaction of spatial restraints. J Mol Biol. 1993;234(3):779–815.

[4] Schrier EE, Schrier EB. The salting-out behavior of amides and its relation to the denaturation of proteins by salts. J Phys Chem. 1967;71(6):1851–1860.

[5] Nandi PK, Robinson DR. Effects of salts on the free energy of the peptide group. J Am Chem Soc. 1972;94(4):1299–1308.

[6] Nandi PK, Robinson DR. Effects of salts on the free energies of nonpolar groups in model peptides. J Am Chem Soc. 1972;94(4):1308–1315.

[7] Baldwin RL. How Hofmeister ion interactions affect protein stability. Biophys J. 1996;71(4):2056–2063.

[8] Kapcha LH, Rossky PJ. A simple atomic-level hydrophobicity scale reveals protein interfacial structure. J Mol Biol. 2014;426(2):484–498.

[9] Müller-Späth S, Sorrano A, Hirschfeld V, Hofmann H, Rüegger S, Reymond L, et al. Charge interactions can dominate the dimensions of intrinsically disordered proteins. Proc Natl Acad Sci USA. 2010;107:14609–14614.

[10] Hofmann H, Soranno A, Borgia A, Gast K, Nettels D, Schuler B. Polymer scaling laws of unfolded and intrinsically disordered proteins quantified with single-molecule spectroscopy. Proc Natl Acad Sci USA. 2012;109:16155–16160.

[11] Sherman E, Haran G. Coil-globule transition in the denatured state of a small protein. Proc Natl Acad Sci USA. 2006;103:11539–11543.

[12] Kjaergaard M, Norholm AB, Hendus-Altenburger R, Pedersen SF, Poulsen FM, Kragelund BB. Temperature-dependent structural changes in intrinsically-disordered proteins: formation of α-helices or loss of polyproline II? Protein Sci. 2010;19:1555–1564.

[13] Flanagan JM, Kataoka M, Shortle D, Engelman DM. Truncated staphylococcal nuclease is compact but disordered. Proc Natl Acad Sci U S A. 1992;89(2):748–752.

[14] Nath A, Sammalkorpi M, DeWitt DC, Trexler AJ, Elbaum-Garfinkle S, OHern CS, et al. The conformational ensembles of α-synuclein and tau: Combining single-molecule FRET and simulations. Biophys J. 2012;103(9):1940–1949.

[15] Kim YC, Hummer G. Coarse-grained models for simulation of multiprotein complexes: application to ubiquitin binding. J Mol Biol. 2008;375:1416–1433.
